# Potential energy landscape formalism for quantum molecular liquids

**DOI:** 10.1038/s42004-024-01342-9

**Published:** 2024-12-04

**Authors:** Ali Eltareb, Yang Zhou, Gustavo E. Lopez, Nicolas Giovambattista

**Affiliations:** 1 Department of Physics, Brooklyn College of the City University of New York, Brooklyn, NY 11210 USA; 2 Ph.D. Program in Physics, The Graduate Center of the City University of New York, New York, NY 10016 USA; 3 Department of Chemistry, Lehman College of the City University of New York, Bronx, NY 10468 USA; 4 Ph.D. Program in Chemistry, The Graduate Center of the City University of New York, New York, NY 10016 USA

**Keywords:** Thermodynamics, Quantum fluids and solids, Computational methods, Statistical mechanics

## Abstract

The potential energy landscape (PEL) formalism is a powerful tool within statistical mechanics to study the thermodynamic properties of classical low-temperature liquids and glasses. Recently, the PEL formalism has been extended to liquids/glasses that obey quantum mechanics, but applications have been limited to atomistic model liquids. In this work, we extend the PEL formalism to liquid/glassy water using path-integral molecular dynamics (PIMD) simulations, where nuclear quantum effects (NQE) are included. Our PIMD simulations, based on the q-TIP4P/F water model, show that the PEL of quantum water is both Gaussian and anharmonic. Importantly, the ring-polymers associated to the O/H atoms in the PIMD simulations, collapse at the local minima of the PEL (inherent structures, IS) for both liquid and glassy states. This allows us to calculate, analytically, the IS vibrational density of states (IS-VDOS) of the ring-polymer system using the IS-VDOS of classical water (obtained from classical MD simulations). The role of NQE on the structural properties of liquid/glassy water at various pressures are discussed in detail. Overall, our results demonstrate that the PEL formalism can effectively describe the behavior of molecular liquids at low temperatures and in the glass states, regardless of whether the liquid/glass obeys classical or quantum mechanics.

## Abstract (web summary)

The potential energy landscape formalism is a powerful tool within statistical mechanics to study the thermodynamic properties of classical low-temperature liquids and glasses. Here, the authors use path-integral molecular dynamics simulations to demonstrate that the formalism can also be used to describe quantum mechanical molecular liquids such as water.

## Introduction

Many theoretical approaches have been proposed to describe the behavior of classical glasses and liquids at low temperatures, close to their glass transition temperature^1–4^. One such approach is the potential energy landscape (PEL) formalism, which is based on classical statistical mechanics^5–8^. The PEL formalism has been used successfully to describe the thermodynamic and dynamical properties of atomistic model liquids^9–11^, atomistic realistic liquids, such as silica^12–14^, and molecular liquids, including orthoterphenyl^15,16^ and water^17–24^. In the case of water, the PEL formalism has been proven to be a valuable method to obtain the corresponding phase diagram at low temperatures where computer simulations are challenging due to the associated slow dynamics^17–20^. Briefly, for a system composed of *d* degrees of freedom {*q*_1_, *q*_3_, …*q*_*d*_}, the PEL is the hypersurface in (*d* + 1)-dimensional space defined by the potential energy of the system as a function of the generalized coordinates, *V*(*q*_1_, *q*_2_, …, *q*_*d*_). Within the PEL formalism, the system is represented by a single point on the PEL that moves with time, describing a path (trajectory) on the PEL. At high temperatures, the system has a large kinetic energy, and it moves rather freely on the PEL, overcoming potential energy barriers and moving from one basin of the PEL to another. At low temperatures, in the glass state, the atoms can only perform vibrational motions, and hence, the representative point of the system can only move within a given basin of the PEL. It follows, that within the PEL formalism, the thermodynamic properties of low-temperature liquids and glasses depend on the topography of the PEL. Indeed, within the PEL formalism, it is possible to express the free energy of the liquid/glass based solely on the statistical properties of the PEL sampled by the system. Specifically, the free energy of the system can be expressed in terms of the average energy of the PEL local minima (inherent structures, IS) sampled by the liquid/glass, the curvature of the PEL about the corresponding IS, and the distribution of IS energies *e*_*IS*_ available in the PEL.

It has been recently shown that using the path-integral formulation of statistical mechanics^25,26^, it is possible to extend the PEL formalism to the case of liquids and glasses that obey quantum mechanics^27,28^. Perhaps the most important difference in the PEL formalism of classical and quantum liquids is that the PEL of a quantum liquid is temperature-dependent, while the PEL of a classical liquid is not. Importantly, computer simulations show that the harmonic and Gaussian approximations of the PEL, which are key in the development of the PEL formalism, may also be extended to the case of quantum liquids. The theoretical predictions of the PEL formalism for a quantum liquid were tested in refs. ^27,28^ with results from path-integral Monte Carlo simulations of a monatomic water-like liquid. While promising, it remains unclear whether the results/conclusions from refs. ^27,28^ are general, applicable to complex molecular liquids. In this work, we build upon the works of refs. ^27,28^ and extend the PEL formalism to the case of water with quantum mechanical effects being included. To do so, we perform PIMD simulations of water using the q-TIP4P/F model^29^, a realistic flexible water model^29–32^, over a wide range of temperatures and pressures, including both liquid and glass states. It is shown that the PEL of quantum water (i.e., as observed in the PIMD simulations) is Gaussian and anharmonic^7,8,33,34^. We note that in PIMD simulations, the water O/H atoms are represented by ring-polymers. Consistent with refs. ^27,28^, our computer simulations show that the ring polymers associated with the O/H atoms collapsed at the IS, i.e., after potential energy minimization of the system. This holds in liquid as well as glassy water, at low and high pressures, including the low-density and high-density amorphous ices (LDA and HDA). As discussed in detail in ref. ^28^, the collapse of the ring-polymers at the IS simplifies enormously the calculation of the vibrational density of states at the IS (IS-VDOS) of the ring-polymer system. Specifically, we confirm that the IS-VDOS of the ring-polymer system associated with quantum water can be calculated analytically from the IS-VDOS of classical water (e.g., obtained from classical MD simulations), as specified in ref. ^28^. We note that even when the ring polymers associated with O/H atoms are collapsed at the IS, they are dispersed in the instantaneous configurations of the liquid and glass states. Indeed, we find that nuclear quantum effects (NQE) are relevant for the thermodynamic and structural properties of q-TIP4P/F water, particularly in the glass state; including NQE (PIMD simulations) are necessary to reproduce the properties of LDA and HDA. Overall, the ultimate goal of this work is to show that a single approach, the PEL formalism, can be used to describe the behavior of realistic molecular liquids and glasses (e.g., water), independently of whether the liquid/glass obeys classical or quantum mechanics.

The structure of this work is as follows. First, we present (i) a brief review of the PEL formalism for a flexible water model treated with quantum mechanics, and (ii) an introduction to the Gaussian and harmonic approximations of the PEL. The results are presented next, followed by a summary and discussion. The computational details are provided in the ‘Methods’ section.

## PEL formalism for a quantum flexible water model

In this section, we discuss the PEL formalism for the case of a flexible water model where the intermolecular interactions depend only on the position of the O and H atoms of the water molecules, which is the case of the q-TIP4P/F water model (the position of the virtual site for a given water molecule is a function of the corresponding O and H atoms coordinates)^29^. Introductions to the PEL formalism for the case of classical liquids are available in the literature (see, e.g., refs. ^7,8,33,35^); the PEL formalism applied to quantum monatomic liquids is discussed in refs. ^27,28^. Here, we follow closely the introduction to the PEL for quantum liquids presented in ref. ^28^. The underlying mathematical expressions are practically the same for quantum monatomic liquids and for water models such as the q-TIP4P/F model; for example, in many expressions, one only needs to replace the number of atoms of the monatomic liquid *N* in ref. ^28^ to *N*→*n* for the case of water, where *n* = 3*N* is the total number of atoms (O and H).

The canonical partition function for a system of *N* water molecules that obey quantum mechanics is given by

1 $$Q(N,V,T)=\,{\mbox{Tr}}\,[\widehat{\rho }]$$

where $\,{\mbox{Tr}}\,(\widehat{\rho })$ is the trace of the density matrix operator $\hat{\rho }=\exp (-\beta \hat{H})$ and

2 $$\hat{H}={\sum }_{i=1}^{n}\frac{{\hat{{{\boldsymbol{p}}}}}_{i}^{2}}{2{m}_{i}}+\hat{U}({\hat{{{\boldsymbol{r}}}}}_{1},{\hat{{{\boldsymbol{r}}}}}_{2},\ldots {\hat{{{\boldsymbol{r}}}}}_{n})$$

is the Hamiltonian operator of the system; $(\hat{{{{\boldsymbol{r}}}}_{i}},\hat{{{{\boldsymbol{p}}}}_{i}})$ are the position and momentum operators associated to atom *i* = 1, 2, …, *n* and *m*_*i*_ is the corresponding mass ($\beta =\frac{1}{{k}_{B}T}$ and *k*_*B*_ is the Boltzmann’s constant).

Using the path-integral formulation of statistical mechanics, one can show that the canonical partition function of the quantum water model is identical to the canonical partition function of a classical system composed of *n*_*b*_ ring-polymers, with peculiar interactions^25,26^. Specifically, it can be shown that Eq. (1) can be written as follows,

3 $$Q(N,V,T)=	 {\lim }_{{n}_{b}\to \infty }\frac{1}{{h}^{3{n}_{b}n}}{\int}_{\!\!\!V}\left[{\prod }_{i=1}^{n}d{{{\boldsymbol{r}}}}_{i}^{1}\ldots d{{{\boldsymbol{r}}}}_{i}^{{n}_{b}}\right]\\ 	 \times \int_{\!\!\!-\infty }^{\infty } \left[{\prod }_{i=1}^{n}d{{{\boldsymbol{p}}}}_{i}^{1}\ldots d{{{\boldsymbol{p}}}}_{i}^{{n}_{b}}\right]\exp \left[-\beta {{{\mathcal{H}}}}_{RP}({{\boldsymbol{R}}},{{\boldsymbol{P}}})\right]$$

where

4 $${{{\mathcal{H}}}}_{RP}({{\boldsymbol{R}}},{{\boldsymbol{P}}})=	 {\sum }_{i=1}^{n}{\sum }_{k=1}^{{n}_{b}}\frac{{({{{\boldsymbol{p}}}}_{i}^{k})}^{2}}{2m{{\prime} }_{i}}+{\sum }_{i=1}^{n}{\sum }_{k=1}^{{n}_{b}}\frac{1}{2}{k}_{i}^{sp}{({{{\boldsymbol{r}}}}_{i}^{k+1}-{{{\boldsymbol{r}}}}_{i}^{k})}^{2}\\ 	+\frac{1}{{n}_{b}}{\sum }_{k=1}^{{n}_{b}}U({{{\boldsymbol{r}}}}_{1}^{k},{{{\boldsymbol{r}}}}_{2}^{k},\ldots ,{{{\boldsymbol{r}}}}_{n}^{k})$$

is the Hamiltonian of the associated classical ring-polymer system. In this expression, $({{{\boldsymbol{r}}}}_{i}^{k},{{{\boldsymbol{p}}}}_{i}^{k})$ are the vector position and momentum of bead *k* = 1, 2, …, *n*_*b*_ of ring-polymer *i* = 1, 2, …, *n*; *n*_*b*_ is the total number of beads per ring-polymer. To simplify the notation, we define ${{\boldsymbol{R}}}=({{{\boldsymbol{r}}}}_{1}^{1},{{{\boldsymbol{r}}}}_{1}^{2},\ldots ,{{{\boldsymbol{r}}}}_{1}^{{n}_{b}};\ldots ;{{{\boldsymbol{r}}}}_{n}^{1},{{{\boldsymbol{r}}}}_{n}^{2},\ldots ,{{{\boldsymbol{r}}}}_{n}^{{n}_{b}})$ and ${{\boldsymbol{P}}}=({{{\boldsymbol{p}}}}_{1}^{1},{{{\boldsymbol{p}}}}_{1}^{2},\ldots ,{{{\boldsymbol{p}}}}_{1}^{{n}_{b}};\ldots ;{{{\boldsymbol{p}}}}_{n}^{1},{{{\boldsymbol{p}}}}_{n}^{2},\ldots ,{{{\boldsymbol{p}}}}_{n}^{{n}_{b}})$. In Eq. (4), ${k}_{i}^{sp}=\frac{{m}_{i}{n}_{b}}{{(\hslash \beta )}^{2}}$ is the (temperature-dependent) spring constant of the ring-polymer *i* associated with atom *i* (O or H) of the system. In this ring-polymer system, only beads with the same index *k* interact with one another. The set of all beads with the same label *k* constitute the so-called replica *k* of the system, and the term $U({{{\boldsymbol{r}}}}_{1}^{k},{{{\boldsymbol{r}}}}_{2}^{k},\ldots ,{{{\boldsymbol{r}}}}_{n}^{k})$ is the total potential energy of replica *k*. In Eq. (4), the mass of the beads belonging to the ring-polymer *i* is given by $m{{\prime} }_{i}={n}_{b}{m}_{i}$, however, the specific value of $m{{\prime} }_{i}$ plays no relevant role in the thermodynamic properties derived from *Q*(*N*, *V*, *T*). Note that in Eq. (4), ${{{\boldsymbol{r}}}}_{i}^{{n}_{b}+1}={{{\boldsymbol{r}}}}_{i}^{1}$ for all *i*, so all the beads belonging to the same ring-polymers form a closed loop (‘ring’).

It follows from Eq. (4), that the potential energy of the ring-polymer system is given by

5 $${{{\mathcal{U}}}}_{RP}({{\boldsymbol{R}}})={\sum }_{i=1}^{n}{\sum }_{k=1}^{{n}_{b}}\frac{1}{2}{k}_{i}^{sp}{({{{\boldsymbol{r}}}}_{i}^{k+1}-{{{\boldsymbol{r}}}}_{i}^{k})}^{2}+\frac{1}{{n}_{b}}{\sum }_{k=1}^{{n}_{b}}U({{{\boldsymbol{r}}}}_{1}^{k},{{{\boldsymbol{r}}}}_{2}^{k},\ldots ,{{{\boldsymbol{r}}}}_{n}^{k})$$

Eq. (5) defines a PEL that can be associated with the quantum water system (for a fixed *n*_*b*_). Next, we apply the PEL formalism to the ring-polymer system defined by Eq. (5). The main idea of the PEL formalism is to partition the PEL into basins^7^. Each basin of ${{{\mathcal{U}}}}_{RP}({{\boldsymbol{R}}})$ is characterized by a local (potential energy) minimum, or inherent structure (IS), and the corresponding basin is defined as the set of points in ${{{\mathcal{U}}}}_{RP}({{\boldsymbol{R}}})$ that converge to the given IS by steepest descent (i.e., upon potential energy minimization). Therefore, each basin of the PEL can be associated with a particular IS characterized by an energy *e*_*IS*_. It can be shown that Eq. (3) can be rewritten as^28^

6 $$Q(N,V,T)={\sum}_{{e}_{IS}}{e}^{-\beta \left[{e}_{IS}-T{S}_{IS}(N,V,T,{e}_{IS})+{F}_{vib}(N,V,T,{e}_{IS})\right]}$$

where the sum runs over all the IS energies available in the PEL. *S*_*IS*_(*N*, *V*, *T*, *e*_*IS*_) is the configurational entropy of the system and it quantifies the number of IS available in the PEL with energy *e*_*IS*_ at the given (*N*, *V*, *T*). Specifically, *S*_*IS*_(*N*, *V*, *T*, *e*_*IS*_) is defined as

7 $${S}_{IS}(N,V,T,{e}_{IS})\equiv {k}_{B}\ln \left[{\Omega }_{IS}(N,V,T,{e}_{IS})\right]$$

where *Ω*_*IS*_(*N*, *V*, *T*, *e*_*IS*_) is the number of IS available in the PEL with energy *e*_*IS*_. Note that in the quantum case, *S*_*IS*_ is a function of (*N*, *V*, *e*_*IS*_) *and T*, unlike in the classical PEL formalism, where *S*_*IS*_ depends only on (*N*, *V*, *e*_*IS*_). *F*_*vib*_(*N*, *V*, *T*, *e*_*IS*_) is the vibrational free energy of the system, i.e., the contribution to the Helmholtz free energy due to the exploration by the system of the PEL about the IS with energy *e*_*IS*_. Specifically,

8 $${F}_{vib}(N,V,T,{e}_{IS})\equiv -{k}_{B}T\ln \left[{ \left\langle {Q}_{l}(N,V,T) \right\rangle }_{{e}_{IS}}\right]$$

where *Q*_*l*_(*N*, *V*, *T*) is the canonical partition function of the system when it is trapped in the basin *l* of the PEL; ${ \langle \ldots \rangle }_{{e}_{IS}}$ indicates averaging over all basins *l* of the PEL with IS energy *e*_*IS*_.

### Single approximation of the PEL formalism

Eq. (6) is exact, but it is of no practical use. As discussed extensively in the literature^7,8,33,35^, in the PEL formalism, one assumes that the system in equilibrium samples a narrow range of *e*_*IS*_-values, which is consistent with numerous computational studies^11,34^, as long as the system remains in only one-phase^10,36^. Equivalently, one applies a saddle-point approximation in Eq. (6), resulting in the following expression,

9 $$Q(N,V,T)\approx {e}^{-\beta \left[{E}_{IS}-T{S}_{IS}(N,V,T,{E}_{IS})+{F}_{vib}(N,V,T,{E}_{IS})\right]}$$

where *E*_*IS*_ = *E*_*IS*_(*N*, *V*, *T*) is the IS energy that maximizes the argument of Eq. (6). Specifically, *E*_*IS*_(*N*, *V*, *T*) is the solution of

10 $$1-T{\left(\frac{\partial {S}_{IS}(N,V,T,{e}_{IS})}{\partial {e}_{IS}}\right)}_{N,V,T}+{\left(\frac{\partial {F}_{vib}(N,V,T,{e}_{IS})}{\partial {e}_{IS}}\right)}_{N,V,T}=0$$

### Gaussian and harmonic approximations for the PEL

In order to proceed further within the PEL formalism, one needs to model the statistical properties of the PEL. The two most commonly used hypothesis in the study of liquids and glasses using the PEL formalism are the (i) Gaussian approximation of the PEL, which assumes that *Ω*_*IS*_(*N*, *V*, *T*, *e*_*IS*_) is a Gaussian distribution, and (ii) the harmonic approximation (HA) of the PEL which assumes that the basins of the PEL have a quadratic shape about the corresponding IS^7,8,33,34^.

(i) In the Gaussian approximation of the PEL, one assumes that

11 $${\Omega }_{IS}(N,V,T,{e}_{IS})=\frac{1}{\sqrt{2\pi {\sigma }^{2}}}{e}^{\alpha N}{e}^{-{({e}_{IS}-{E}_{0})}^{2}/2{\sigma }^{2}}$$

where, for quantum liquids^28^, (*α*, *σ*^2^, *E*_0_) are PEL variables that depend on (*N*, *V*, *T*). At a given (*N*, *V*, *T*), the total number of IS in the PEL is given by *e*^*αN*^, and the average IS energy and variance of *Ω*_*IS*_(*N*, *V*, *T*, *e*_*IS*_) are given by *E*_0_ and *σ*^2^, respectively. Using Eq. (11) in Eq. (7), one finds that

12 $${S}_{IS}(N,V,T,{e}_{IS})\approx {k}_{B}\left[\alpha N-\frac{{({e}_{IS}-{E}_{0})}^{2}}{2{\sigma }^{2}}\right]$$

(ii) In the HA, the vibrational free energy *F*_*vib*_ can be calculated analytically (using Eq. (8))^28,33^. Specifically, one finds that

13 $${F}_{vib}(N,V,T)\approx d{n}_{b}{k}_{B}T\ln (\beta {A}_{0})+{k}_{B}T{{\mathcal{S}}}(N,V,T,{e}_{IS})$$

where *d* is the number of degrees of freedom in the system (*d* = 9*N* for q-TIP4P/F water) and ${{\mathcal{S}}}(N,V,T,{e}_{IS})$ is the so-called basin shape function^33^. ${{\mathcal{S}}}(N,V,T,{e}_{IS})$ quantifies the average local curvature of the basins with IS energy equal to *e*_*IS*_, about the corresponding IS, and is given by

14 $${{\mathcal{S}}}(N,V,T,{e}_{IS})={\left\langle {\sum }_{i = 1}^{d{n}_{b}-3}\ln \left(\frac{\hslash {\omega }_{i}(N,V,T,{e}_{IS})}{{A}_{0}}\right)\right\rangle }_{{e}_{IS}}$$

In this expression, the *T*-dependent *dn*_*b*_ values $\{{\omega }_{i}^{2}\}$ are the eigenvalues of the *mass-weighted* Hessian matrix of the ring-polymer system evaluated at temperature *T*, at the IS of the PEL with energy *e*_*IS*_ (all *dn*_*b*_ eigenvalues are greater than zero, except for 3 zero eigenvalues, which correspond to the three independent translations of the system center of mass); ${ \langle \ldots \rangle }_{{e}_{IS}}$ indicates an average over all basins of the PEL with energy *e*_*IS*_, and *A*_0_ ≡ 1 kJ/mol is a constant that ensures that the argument of the $\ln (\ldots )$ has no units. As previously found in ref. ^28^, we find that the shape function of the q-TIP4P/F water model is linear with *e*_*IS*_ (see supplementary Note 5),

15 $${{\mathcal{S}}}(N,V,T,{e}_{IS})=a(N,V,T)+b(N,V,T)\,{e}_{IS}$$

where *a* and *b* are coefficients that depend on (*N*, *V*, *T*). A similar relationship is found in classical atomistic and molecular systems; however, for classical systems, *a* and *b* are functions of only (*N*, *V*)^15,17,18,21,37^.

Within the Gaussian and harmonic approximation of the PEL (Eqs. (11) and (13)), Eq. (10) can be solved, resulting in the following expression for *E*_*IS*_,

16 $${E}_{IS}(N,V,T)={E}_{0}-{\sigma }^{2}(b+\beta )$$

where, again, *E*_0_, *b*, and *σ*^2^ all depend on (*V*, *T*). We stress that in the classical case, the PEL variables {*E*_0_, *σ*^2^, *α*} are functions of only *V*^28^.

*Helmholtz free energy of water with a Gaussian and harmonic PEL*. The Helmholtz free energy of the system, $F(N,V,T)=-{k}_{B}T\ln [Q(N,V,T)]$, follows directly from Eq. (9),

17 $$F(N,V,T)={E}_{IS}(N,V,T)-T{S}_{IS}(N,V,T,{E}_{IS})+{F}_{vib}(N,V,T,{E}_{IS})$$

In addition, if the PEL of quantum water is Gaussian and harmonic, one can obtain all of the thermodynamic properties of the quantum liquid using Eq. (17). For example, as shown in ref. ^28^, the energy of the quantum liquid is given by $E(N,V,T)={\left[\partial (\beta F)/\partial \beta \right]}_{N,V}$ which, using Eq. (17), can be written as

18 $$E={E}_{IS}\,+\,d{n}_{b}{k}_{B}T+\left[{\left(\frac{\partial {{\mathcal{S}}}}{\partial \beta }\right)}_{N,V,{E}_{IS}}-N{\left(\frac{\partial \alpha }{\partial \beta }\right)}_{V}+(\beta +b){\left(\frac{\partial {E}_{0}}{\partial \beta }\right)}_{V}-\frac{{(\beta +b)}^{2}}{2}{\left(\frac{\partial {\sigma }^{2}}{\partial \beta }\right)}_{V}\right]$$

Importantly, the vibrational energy *E*_*vib*_ ≡ *E*−*E*_*IS*_ is given by (Gaussian and harmonic approximations of the PEL),

19 $${E}_{vib}\to {E}_{vib}^{harm}=d{n}_{b}{k}_{B}T+\left[{\left(\frac{\partial {{\mathcal{S}}}}{\partial \beta }\right)}_{N,V,{E}_{IS}}-N{\left(\frac{\partial \alpha }{\partial \beta }\right)}_{V}+(\beta +b){\left(\frac{\partial {E}_{0}}{\partial \beta }\right)}_{V}-\frac{{(\beta +b)}^{2}}{2}{\left(\frac{\partial {\sigma }^{2}}{\partial \beta }\right)}_{V}\right]$$

Eq. (19) is the vibrational energy associated with the *quantum liquid* in the PEL formalism, and it is related to the exploration, by the ring-polymer system, of the basins of the RP-PEL. However, *E*_*vib*_, as well as *E*_*IS*_, have no clear physical meaning from the quantum liquid perspective (if any). We also note that Eq. (19) holds for *classical* water. When applied to the classical case (*n*_*b*_ = 1 and […] = 0), Eq. (19) reduces to the corresponding classical expression, ${E}_{vib}^{harm}=d{k}_{B}T$^18^. It follows that the brackets in Eqs. (18) and (19) are unique to the quantum water system because, in the quantum case, the PEL is *T*-dependent. As we will discuss below, in the case of q-TIP4P/F water, it is possible to neglect the *T*-dependence of the PEL variables (*α*, *E*_0_, *σ*^2^), simplifying Eq. (19). This implies that, while the RP-PEL is *T*-dependent, the distribution of the IS in the RP-PEL is not.

## Results

The results are presented as follows. We first discuss the main properties of the RP-PEL associated with q-TIP4P/F water. Specifically, we study the temperature-dependence of the IS energy *E*_*IS*_(*T*), vibrational energy *E*_*vib*_(*T*), IS vibrational density of state, and shape function ${{\mathcal{S}}}(T)$ of the RP-PEL. Then, we test whether the harmonic and Gaussian approximations apply to the RP-PEL. We conclude with a discussion of the properties of the RP-PEL sampled by q-TIP4P/F water during the vitrification (isobaric cooling) into LDA and HDA.

### IS and vibrational energy

**Fig. 1:**
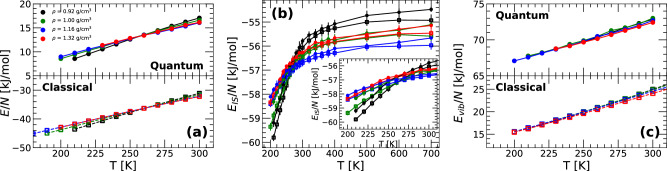
Comparison of the thermodynamic and PEL properties obtained from classical MD and PIMD simulations of q-TIP4P/F water. **a** Total energy *E*(*T*), **b** IS energy *E*_*IS*_(*T*), and **c** vibrational energy *E*_*vib*_(*T*) ≡ *E*(*T*)−*E*_*IS*_(*T*) obtained from classical MD (empty squares) and PIMD simulations (solid circles) at selected densities. Introducing NQE (PIMD simulations) increases considerably the total and vibrational energies of q-TIP4P/F water. Instead, the *E*_*IS*_(*T*) of q-TIP4P/F water changes very weakly when NQE is included/excluded [see inset in **b**].

Figure 1 shows the (a) total energy *E*(*T*), (b) IS energy *E*_*IS*_(*T*), and (c) vibrational energy *E*_*vib*_(*T*) ≡ *E*(*T*)−*E*_*IS*_(*T*) of q-TIP4P/F water as a function of temperature obtained from PIMD simulations at selected densities. For comparison, also included in Fig. 1 are the *E*(*T*), *E*_*IS*_(*T*), and *E*_*vib*_(*T*) of q-TIP4P/F water obtained from classical MD simulations (*n*_*b*_ = 1) from ref. ^18^.

The behavior of *E*(*T*) for q-TIP4P/F water obtained from MD and PIMD simulations are qualitatively similar. However, PIMD simulations predict much larger total energies *E*(*T*) for q-TIP4P/F water than MD simulations. This is because PIMD simulations incorporate NQE, including zero-point energies, which represent a non-negligible contribution to the OH-covalent bond and HOH angle bending potential energies. In terms of the PEL formalism, the origin of the different values of *E*(*T*) can be tracked down mostly to the vibrational energy *E*_*vib*_(*T*). Specifically, *E*(*T*) = *E*_*IS*_(*T*) + *E*_*vib*_(*T*) and, as shown in Fig. 1b, the value of *E*_*IS*_(*T*) from MD and PIMD simulations are very similar (typical differences in *E*_*IS*_ are *δE*_*IS*_ < 1 kJ/mol). Accordingly, the differences in the total energy of q-TIP4P/F water when NQE are/are not included arise mainly due to the larger vibrational energy *E*_*vib*_(*T*) when NQE are included [Fig. 1c]. It follows from Eq. (19), that the large values of *E*_*vib*_(*T*), when NQE are included, are due to the introduction of ‘beads’ (*n*_*b*_ > 1), which is inherent to the path-integral formalism of statistical mechanics, and to the large values of ${\left(\partial {{\mathcal{S}}}/\partial T\right)}_{N,V,{E}_{IS}}$ (see section ‘Testing the Harmonic Approximation of the PEL’).

The behavior of *E*_*IS*_(*T*) calculated from MD and PIMD simulations is particularly interesting. Fig. 1b indicates that independently of whether one includes/excludes NQE, *E*_*IS*_(*T*) is constant at large temperatures, in the so-called PEL-independent regime, where the topography of the PEL is not relevant^9^. Moreover, at low temperatures (~*T* < 300 K), *E*_*IS*_(*T*) decreases upon cooling, in the so-called PEL-influenced regime, where the topographic properties of the PEL play a major role^9^. The crossover temperature between the PEL-influenced and PEL-independent regimes occurs at the onset temperature *T*_0_^9,33^. It follows from Fig. 1b that the onset temperature for the ring-polymer system associated with q-TIP4P/F water is *T*_0_ = 280−330 K^18^, depending on the density, and it is practically independent of whether one includes NQE. Similar values of *T*_0_ are found in classical MD simulations studies of different water models^17,19^. In previous studies^27,28^, we explored the PEL of an atomistic quantum model liquid using PIMD simulations. In agreement with the results for q-TIP4P/F water, it was found that the monatomic quantum liquid was also characterized by PEL-independent and PEL-influenced regimes. In particular, it was found that including NQE shifted *E*_*IS*_(*T*) (and *T*_0_) to lower temperatures. As shown in the inset of Fig. 1b, including NQE also shift *E*_*IS*_(*T*) towards lower temperatures. However, the corresponding shift in temperature is very weak, observable at low temperatures, and it varies with the density considered.

### Vibrational density of states of the ring-polymer system at the IS

In this section, we first show that the ring-polymers associated with q-TIP4P/F water collapse at the IS, i.e., during minimization of the potential energy of the system. Similar conclusions were reported in refs. ^27,28^ for the case of a water-like monatomic liquid, using PIMD simulations. As explained in detail in ref. ^28^, the collapse of the ring-polymers at the IS represent an enormous simplification of the PEL formalism when applied to the study of quantum liquids/glasses. Specifically, for IS with collapsed ring-polymers, one can show that the normal modes of the ring-polymer system at the IS of the RP-PEL can be obtained analytically from the normal modes of the corresponding *classical* liquid at the IS of the CL-PEL^28^. For example, in the case of a 3D-system composed of *N* identical atoms, the eigenvalues of the *mass-weighted* Hessian matrix of the quantum liquid, $\{{\omega }_{i,j}^{2}\}$ (*i* = 1, 2, …, *N* and *j* = 1, 2, …, *n*_*b*_) can be obtained analytically from the eigenvalues of the *mass-weighted* Hessian matrix of the classical liquid, $\{{\omega }_{i,0}^{2}\}$ (*i* = 1, 2, …, 3*N*) as follows (see ref. ^28^ and Supplementary Note 1 of the supplementary material (SM)),

20 $${\omega }_{i,j}^{2}=\frac{{\omega }_{i,0}^{2}}{{n}_{b}^{2}}-\frac{2}{{(\hslash \beta )}^{2}}\left[\cos \left(\frac{2\pi }{{n}_{b}}j\right)-1\right]$$

#### Collapse of the ring polymers at the IS

We focus on the radius of gyration *R*_*g*_ of the ring polymers representing the O/H atoms of q-TIP4P/F water. Specifically,

21 $${R}_{g}=\left\langle \frac{1}{{n}_{b}}{\sum }_{j=1}^{{n}_{b}}{({{{\boldsymbol{r}}}}_{c}-{{{\boldsymbol{r}}}}_{j})}^{2}\right\rangle$$

where ***r***_*c*_ is the center of mass (centroid) of the given ring-polymer and ***r***_*j*_ is the position of the ring-polymer’s bead *j* = 1, 2, …, *n*_*b*_; $\langle \ldots \rangle$ indicates an average over time and all ring-polymers in the system (associated to the same kind of atom, i.e., O or H). *R*_*g*_ is a standard property used in PIMD simulations to quantify the quantum delocalization of atoms due to quantum fluctuations. Indeed, it can be shown within the PI formalism of statistical mechanics that the *R*_*g*_ of a given ring-polymer is related to the quantum mechanics uncertainty in the position of the corresponding atom.

**Fig. 2:**
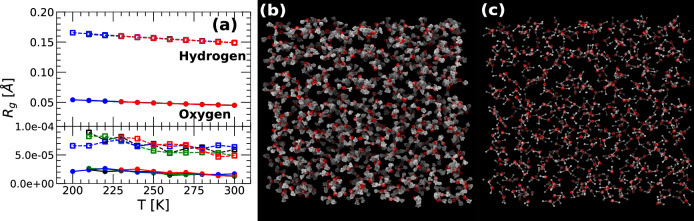
Atoms delocalization of q-TIP4P/F water at the instantaneous configurations and corresponding IS. **a** Radius of gyration, *R*_*g*_(*T*), of the ring-polymers associated to the water O (circles) and H (squares) atoms. *R*_*g*_(*T*) is calculated at the instantaneous configurations sampled during the PIMD simulations (upper panel) and at the corresponding IS (lower panel). In the instantaneous configurations, *R*_*g*_(*T*) increases weakly upon cooling, i.e., the delocalization of the H/O atoms increases slightly with decreasing temperature. Instead, at the IS, ${R}_{g}^{IS}(T)\approx 0$ implying that all ring-polymers are collapsed. **b** Snapshot of an instantaneous configuration of the system at *ρ* = 1.00 g/cm^3^ and *T* = 240 K. **c** Snapshot of the system at the IS corresponding to the configuration shown in **b**.

The *R*_*g*_(*T*) of the O and H atoms of q-TIP4P/F water, as a function of temperature, are shown in Fig. 2a for the instantaneous configurations (upper panel) and the corresponding IS (lower panel). In the instantaneous configurations, the values of *R*_*g*_(*T*) for both the O and H atoms increase upon cooling, implying that the atoms become increasingly delocalized with decreasing temperatures. This is consistent with previous PIMD computer simulations of liquid/glassy water^38^ and water-like atomistic models^39^. However, at the temperatures studied in this work, where water is in the liquid state (*T* = 200–300 K), the changes in *R*_*g*_(*T*) for the O and H atoms are rather small. Interestingly, our PIMD simulations also indicate that the O/H *R*_*g*_(*T*) does not vary with density. This is also consistent with PIMD simulations of a monatomic quantum liquid which shows a very mild density-dependence of *R*_*g*_ at constant temperature^40^.

The most important point of Fig. 2a is that, at the IS, the radius of gyration of the H and O atoms is practically zero, ${R}_{g}^{IS}(T)\approx 1{0}^{-5}-1{0}^{-4}$ Å, at all temperatures and densities studied; in other words, the ring-polymers are collapsed at the IS. A snapshot of q-TIP4P/F water obtained from PIMD simulations at *T* = 240 K and *ρ* = 1.00 g/cm^3^ is included in Fig. 2b, where the delocalization of the O and, particularly, the H atoms can be observed. Fig. 2c shows the system at the IS corresponding to the configuration shown in Fig. 2b; in this case, the O and H atoms are fully localized. As explained in refs. ^27,28^, the collapse of the ring-polymers at the IS implies that the IS of the PEL associated with the ring-polymer system (RP-PEL) are also IS of the PEL associated with the *classical* liquid (*n*_*b*_ = 1) (CL-PEL).

One may wonder whether the ring-polymers associated with the O/H atoms of water may also collapse at the IS obtained at very low temperatures. To address this question, we cool (vitrify) liquid water from *T* = 240 K down to 40 K at *P* = 0.1 and 1000 MPa (cooling rate *q*_*T*_ = 10 K/ns) and calculate the IS visited by the system during the process. At *P* = 0.1 MPa and very low temperatures, q-TIP4P/F water transforms to a LDA^38^; similarly, at *P* = 1000 MPa and very low temperatures, water transforms to a HDA^41^. As shown in section ‘Inherent Structures of q-TIP4P/F Water Sampled During Vitrification at *P* = 0.1, 1000 MPa’, during the cooling process, the *R*_*g*_(*T*) of the O/H atoms increase monotonically with decreasing temperatures. Yet, at the IS, ${R}_{g}^{IS}(T)\approx 0$ at all the temperatures studied (including the LDA and HDA states).

#### Inherent structure vibrational density of states

**Fig. 3:**
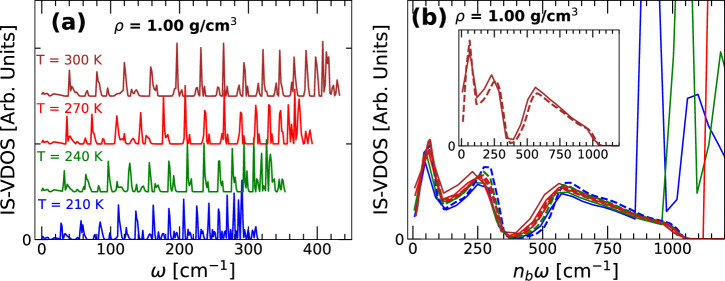
Vibrational density of states of the ring-polymer system associated to q-TIP4P/F water evaluated at the inherent structures (IS-VDOS). **a** IS-VDOS at selected temperatures and density *ρ* = 1.00 g/cm^3^. Consistent with ref. ^28^, the IS-VDOS shifts towards low frequencies as the temperature decreases. **b** Magnification of the IS-VDOS shown in **a** at low frequencies (~*ω* < 1200/*n*_*b*_ cm^−1^). Inset: Comparison of the IS-VDOS of quantum (solid line; *n*_*b*_ = 32) and classical (dashed line; *n*_*b*_ = 1) q-TIP4P/F water at *T* = 300 K. In all cases, the IS-VDOS is calculated using Eq. (20) (see text).

Calculating the vibrational density of states at the IS (IS-VDOS) for the ring-polymer system associated with the q-TIP4P/F water system is computationally challenging. Even for our modest system size (*N* = 512 water molecules and *n* = 32), we could not calculate the IS-VDOS numerically due to the excessive computer time needed for the calculation. The Hessian matrix for such a system is a (9*Nn*_*b*_ × 9*Nn*_*b*_)-square matrix. While calculating the Hessian matrix is doable, the diagonalization of such a matrix is beyond our computational resources. Accordingly, the IS-VDOS for q-TIP4P/F water is evaluated analytically using Eq. (20) (as shown in Supplementary Note 1, the eigenvalues of the *mass-weighted* Hessian matrix (vibrational density of states) at the IS of the RP-PEL associated to q-TIP4P/F water are also given by Eq. (20)). Eq. (20) is validated in the SM by calculating the IS-VDOS of small clusters of water molecules. Specifically, we find that Eq. (20) is in excellent agreement with the IS-VDOS calculated numerically for small clusters composed of *N* = 4, 16, 32, 96 water molecules [see Supplementary Note 4 of the SM]. Figure 3 shows the IS-VDOS of the ring-polymer system associated with q-TIP4P/F water obtained from Eq. (20). To do so, we perform classical MD simulations of q-TIP4P/F water (*n*_*b*_ = 1) to get the frequencies *ω*_*i*,0_ for the classical system and then, use Eq. (20) to calculate the frequencies *ω*_*i*,*j*_ of the quantum version of q-TIP4P/F water.

Figure 3a, b show the IS-VDOS at a fix density, *ρ* = 1.00 g/cm^3^, and for different temperatures. As explained in ref. ^28^, the IS-VDOS of the ring-polymer system is relevant within the PEL formalism and is not evidently related to the (physically relevant) VDOS of the real liquid. The IS-VDOS of the quantum liquid is composed of (i) large peaks located at high frequencies that shift considerably with temperature. These normal mode frequencies are due to the ring-polymer springs and correspond to the frequencies given by Eq. (20) for *j* < *n*_*b*_. The IS-VDOS also contains (ii) low frequencies that correspond to the normal mode frequencies of the classical liquid. These normal mode frequencies are independent of the ring-polymer springs, and are given by Eq. (20) with *j* = *n*_*b*_. Indeed, the IS-VDOS of the classical liquid is included in the IS-VDOS of the corresponding quantum liquid, with the frequencies re-scaled by 1/*n*_*b*_ [from Eq. (20), ${\omega }_{i,j = {n}_{b}}={\omega }_{i,0}/{n}_{b}$ for *i*  = 1, 2, …9*N*]. As discussed below, our results in Fig. 3 are in full agreement with points (i) and (ii).

In the case of q-TIP4P/F water, the IS-VDOS obtained from classical MD simulations (*n*_*b*_ = 1) extends up to *ω* ≈ 3800 cm^−1^ ^18^. Hence, the low frequencies corresponding to the point (ii) in Fig. 3a, b are located at approximately *ω* < 3800/*n*_*b*_ cm^−1^. Indeed, the IS-VDOS shown in Fig. 3b at *T* = 300 K for *ω* < 1200/*n*_*b*_ cm^−1^ are identical to the normal mode frequencies of the classical q-TIP4P/F water re-scaled by 1/*n*_*b*_ (see inset). The translational modes of the classical q-TIP4P/F water correspond to approximately *ω* < 400/*n*_*b*_ cm^−1^; the normal modes for 400/*n*_*b*_ < *ω* < 1200/*n*_*b*_ cm^−1^ correspond to the librational normal mode frequencies of the classical q-TIP4P/F water [the bending HOH and stretching OH modes are located at *ω* ≈ 1600/*n*_*b*_ and *ω* ≈ 3800/*n*_*b*_, respectively, and are not visible in Fig. 3b]. As explained in ref. ^28^, in these low-frequency vibrational modes [point (ii)], the q-TIP4P/F water molecules oscillate in the same manner as the classical q-TIP4P/F water counterparts, with the ring-polymers collapsed at all times, and with lower frequency ${\omega }_{i,j = {n}_{b}}={\omega }_{i,0}/{n}_{b}$ (*i* = 1, 2, …9*N*).

The large peaks in the IS-VDOS shown in Fig. 3b and at ~*ω* > 1100/*n*_*b*_ cm^−1^ correspond to the high-frequency normal modes described in point (i) above. These frequencies shift considerable toward lower values of *ω* upon cooling [Fig. 3a]. As shown in Fig. 3b, at the lower temperatures studied (*T* = 210 K), these high-frequency peaks in the IS-VDOS overlap with the low frequencies translational and librational modes associated to the classical q-TIP4P/F water, located at *ω* < 1100/*n*_*b*_ cm^−1^. We find that at even lower temperatures, in the LDA and HDA states, the normal mode frequencies corresponding to points (i) and (ii) completely overlap; see section ‘Inherent Structures of q-TIP4P/F Water Sampled During Vitrification at *P* = 0.1, 1000 MPa’.

#### Shape function

The shape function ${{\mathcal{S}}}(N,V,T,{E}_{IS})$ is an important property of the PEL formalism that quantifies the curvature of the PEL about the IS sampled by the system at a given working conditions, (*N*, *V*, *T*). The shape function can be calculated in a straightforward manner if the IS-VDOS of the system is known; in this work, we calculate the ${{\mathcal{S}}}$ of the PEL associated to q-TIP4P/F water using Eq. (14) and the IS-VDOS calculated above. Overall, the results presented here are fully consistent with the MC/PIMC simulations of ref. ^28^ based on a monatomic water-like model liquid.

**Fig. 4:**
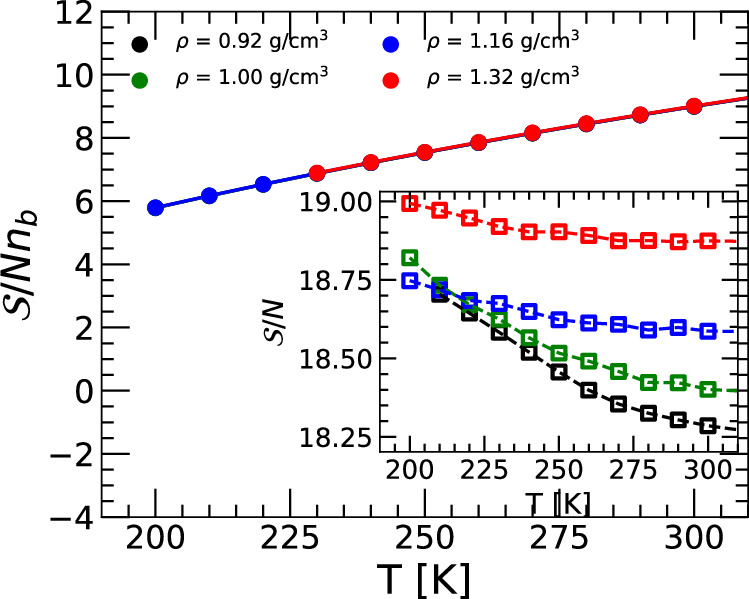
Shape function ${{\mathcal{S}}}(T)$ about the IS of the ring-polymer system associated to q-TIP4P/F water. Temperature-dependence of the shape function ${{\mathcal{S}}}(T)$ of the ring-polymer system associated with q-TIP4P/F water. ${{\mathcal{S}}}(T)$ is evaluated at the IS of the ring-polymer PEL sampled by the system at different temperatures and densities. The inset shows the ${{\mathcal{S}}}(T)$ of the PEL of q-TIP4P/F water obtained from classical MD simulations reported in ref. ^18^ (*n*_*b*_ = 1). For a better comparison, in the main panel, ${{\mathcal{S}}}(T)$ is normalized by the number of beads per ring-polymer *n*_*b*_. While in the classical case ${{\mathcal{S}}}(T)$ increases weakly upon cooling, including NQE leads to an ${{\mathcal{S}}}(T)$ that decreases strongly with decreasing temperatures.

Figure 4 shows the ${{\mathcal{S}}}$ for the ring-polymer system associated to q-TIP4P/F water as a function of temperature, for selected densities. The inset of Fig. 4 shows the ${{\mathcal{S}}}$ of the classical q-TIP4P/F water, obtained from MD simulations. In classical q-TIP4P/F water, ${{\mathcal{S}}}(T)$ increases very weakly upon cooling, indicating that the basins of the CL-PEL become slightly thinner with decreasing temperature (inset). Instead, including NQE leads to a ${{\mathcal{S}}}$ for q-TIP4P/F water that decreases upon cooling, i.e., the basins of the RP-PEL about the IS become wider with decreasing temperature. Interestingly, the inset of Fig. 4 shows weak density effects on the shape function of the classical q-TIP4P/F water, with $\delta {{\mathcal{S}}} < 0.50$. We note that similar density effects (with $\delta {{\mathcal{S}}} < 0.50$) are also found for the quantum q-TIP4P/F water. However, such differences in ${{\mathcal{S}}}$ are not visible in the main panel of Fig. 4 due to the large scale of the *y* axis and the corresponding prefactor 1/*n*_*b*_.

**Fig. 5:**
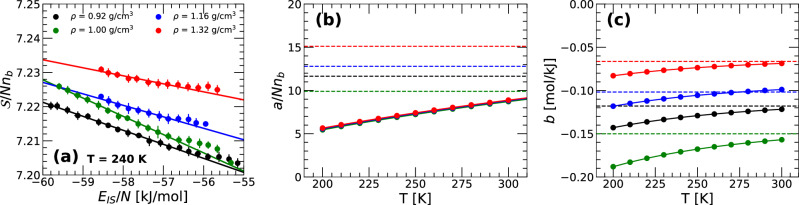
Shape function ${{\mathcal{S}}}({E}_{IS})$ of the ring-polymer system associated with q-TIP4P/F water as a function of *E*_*IS*_. **a** ${{\mathcal{S}}}({E}_{IS})$ (divided by *n*_*b*_) for selected densities. Solid circles correspond to the ${{\mathcal{S}}}({E}_{IS})$ obtained by using Eq. (14) and the corresponding IS-VDOS (see, e.g., Fig. 3); solid lines are given by Eq. (22) with the PEL variables *a*(*T*) and *b*(*T*) given in **b**, **c**. For comparison, also included in **b**, **c** are the (constant) values of *a* and *b* obtained from classical MD simulations of q-TIP4P/F water^18^ (dashed lines). Note that in **b**, the PEL variable *a*(*T*) are scaled by the factor 1/*n*_*b*_.

Computational studies of the PEL based on classical atomistic and molecular model liquids^11,15,16,37^, including water^17,18,21,42^, show that, in the absence of NQE, ${{\mathcal{S}}}(N,V,{e}_{IS})$ has a simple dependence on *e*_*IS*_. Specifically, at constant *N* and *V*,

22 $${{\mathcal{S}}}({e}_{IS})=a+b\,{e}_{IS}$$

where *a* and *b* depend only on (*N*, *V*) [note that in equilibrium, *e*_*IS*_→*E*_*IS*_; see Eq. (10)]. In ref. ^28^, it is found that Eq. (22) also holds for the RP-PEL associated with a quantum monatomic model liquid. However, since for quantum liquids the PEL is *T*-dependent [i.e., ${{\mathcal{S}}}={{\mathcal{S}}}(N,V,T;{e}_{IS})$], the PEL variables *a* and *b* in Eq. (22) are functions of (*N*, *V*, *T*). We test whether Eq. (22) also holds for q-TIP4P/F water when NQE are included (with *a* and *b* being *T*-dependent). To do so, we follow the same procedure employed in ref. ^28^ to calculate ${{\mathcal{S}}}(T;{e}_{IS})$ for each density studied (with constant *N* and *V*; see also Supplementary Note 5 of the SM). As shown in the SM, our calculations at selected temperatures show that, indeed, Eq. (22) holds for q-TIP4P/F water with *a* = *a*(*T*) and *b* = *b*(*T*) (for given *N* and *V*). As an example, included in Fig. 5a are the values of ${{\mathcal{S}}}(T;{e}_{IS})$ as a function of *e*_*IS*_ obtained at *T* = 240 K and different densities. The lines in Fig. 5a correspond to the expression in Eq. (22). The PEL variables *a*(*T*) and *b*(*T*) for q-TIP4P/F water, at a given density (*N* = 512), are shown in Fig. 5b, c. For comparison, also included are the results obtained from ref. ^18^ for the classical q-TIP4P/F water based on MD simulations (dashed lines). The values of *b*(*T*) from MD and PIMD simulations are comparable in magnitude but including NQE leads to a *b*(*T*) that varies weakly with temperature. Instead, the values of *a*(*T*) are much larger when NQE is included. This is due to the large normal mode frequencies of the ring-polymer system (see Eqs. (14) and (20)). Overall, including NQE leads to a modest *T*-dependence of *a*(*T*).

### Testing the harmonic approximation of the PEL

In this section, we show that the harmonic approximation of the PEL does not hold for the case of q-TIP4P/F water. As for the case of classical water models^17–19^, anharmonicites need to be included to describe the properties of q-TIP4P/F water. To test whether the HA holds for q-TIP4P/F water, including NQE, we evaluate the vibrational energy *E*_*vib*_ ≡ *E*−*E*_*IS*_ from PIMD simulations and compare it with the predictions from the PEL formalism using the harmonic approximation,

23 $${E}_{vib}^{harm}(N,V,T)=d{n}_{b}{k}_{B}T+{\left(\frac{\partial {{\mathcal{S}}}}{\partial \beta }\right)}_{N,V,{E}_{IS}}$$

Eq. (23) corresponds to Eq. (19) with the further assumptions that the PEL variables *α*, *E*_0_, and *σ*^2^ do not depend on *T* (they are only functions of *V*); see ref. ^28^. Eq. (23) requires one to calculate ${(\partial {{\mathcal{S}}}/\partial \beta )}_{N,V,{E}_{IS}}$ which follows directly from Eq. (22).

**Fig. 6:**
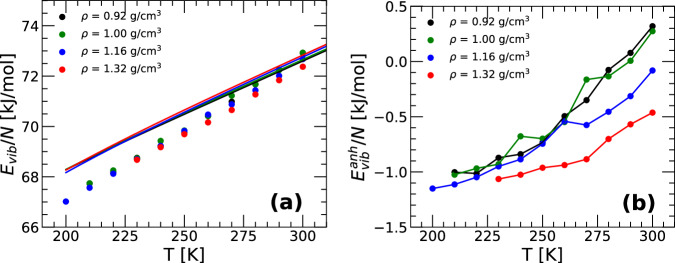
Vibrational energy of q-TIP4P/F water within the PEL formalism. **a** *E*_*vib*_(*T*) as a function of temperature at selected densities. Circles correspond to results obtained from PIMD simulations; lines are the corresponding predictions based on the harmonic approximation of the PEL, ${E}_{vib}^{harm}$ (Eq. (23)). For all the densities considered, the PIMD simulation results are in relatively good agreement with Eq. (23) but anharmonic corrections are non-negligible. **b** Anharmonic contributions to the vibrational energy, ${E}_{vib}^{anh}\equiv {E}_{vib}-{E}_{vib}^{harm}$ obtained from (a).

Figure 6a shows *E*_*vib*_(*T*) for q-TIP4P/F water obtained from PIMD simulations (solid circles) at selected densities. The predictions from Eq. (23) based on the harmonic approximation of the PEL correspond to the lines in the figure. The agreement between PIMD simulations and the PEL approach is relatively good for all temperatures and densities, but deviations between the PIMD simulations and Eq. (23) are non-negligible. The deviations in *E*_*vib*_ from its harmonic approximation are quantified by the corresponding anharmonic vibrational energy ${E}_{vib}^{anh}(T)\equiv {E}_{vib}(T)-{E}_{vib}^{harm}(T)$ and is included in Fig. 6b. We note that PEL studies of classical water models^17,19^ including the classical version of the q-TIP4P/F model^18^, show that anharmonicites are necessary to describe the thermodynamics properties of water. In this regard, the values of ${E}_{vib}^{anh}(T)$ shown in Fig. 6b are comparable in magnitude to the corresponding anharmonic corrections reported in classical water models, including the SPC/E, TIP4P/2005, and (classical) q-TIP4P/F models. Overall, the agreement between Eq. (23) and the PIMD simulations is consistent with the behavior found in a quantum water-like monatomic liquid in ref. ^28^, providing support that Eq. (23) can be used for realistic (complex) molecular liquids.

### Gaussian approximation

**Fig. 7:**
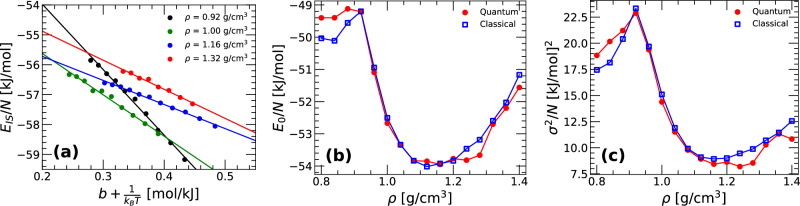
Evidence showing that the PEL of the ring-polymer system associated to q-TIP4P/F water is Gaussian. **a** Inherent structure energy *E*_*IS*_(*T*) obtained from PIMD simulations of q-TIP4P/F water as a function of $[b(T)+\frac{1}{{k}_{B}T}]$ for different densities (red circles). At *T* ≤ 280 K, *E*_*IS*_(*T*) is a linear function of $[b(T)+\frac{1}{{k}_{B}T}]$ consistent with the Gaussian approximation of the PEL, Eq. (16). **b**, **c** PEL variables *E*_0_(*V*) and *σ*^2^(*V*) as a function of density obtained from the linear fits in **a** using Eq. (16). For comparison, also included in **b**, **c** are the values of *E*_0_(*V*) and *σ*^2^(*V*) reported for classical q-TIP4P/F water (blue squares) from ref. ^18^. The values of *E*_0_(*V*) and *σ*^2^(*V*) for the classical and ring-polymer PEL are very similar and show the same qualitative dependence.

In this section, we show that the Gaussian approximation of the PEL is consistent with results obtained from PIMD simulations of q-TIP4P/F water. To show this, we compare the IS energy *E*_*IS*_(*T*) calculated from the PIMD simulations performed at different densities with the prediction from the PEL formalism using the Gaussian approximation, Eq. (16). Figure 7a shows the average IS energy *E*_*IS*_(*T*) as a function of $[b(T)+\frac{1}{{k}_{B}T}]$ for selected densities. The main point of Fig. 7a is that, for all densities considered, ${E}_{IS}(T)\propto [b(T)+\frac{1}{{k}_{B}T}]$ for *T* < 280 K, consistent with the prediction of Eq. (16). We note that, in the quantum PEL formalism, the PEL variable *b*(*T*) depends on *T*, which is not the case for classical liquids. While *E*_0_ and *σ*^2^ may also depend on *T* for a quantum liquid, throughout this work we assume that these PEL variables are *T*-independent. The parameters *E*_0_(*V*) and *σ*^2^(*V*) for the quantum q-TIP4P/F water are evaluated from Fig. 7a by fitting the PIMD simulations data (circles) to Eq. (16). *E*_0_(*V*) and *σ*^2^(*V*) are shown in Fig. 7b, c. For comparison, we also include the values of *E*_0_(*V*) and *σ*^2^(*V*) for classical q-TIP4P/F (*n*_*b*_ = 1; blue open squares) reported in ref. ^18^. The values and volume-dependence of *E*_0_(*V*) and *σ*^2^(*V*) for quantum q-TIP4P/F water are remarkably similar to the corresponding values for classical q-TIP4P/F water. This suggests that the inclusion of NQE does not necessarily affect the distribution of IS in the PEL, *Ω*_*IS*_(*N*, *V*, *T*, *e*_*IS*_) (up to the factor $\exp [N\,\alpha (V)]$; see Eq. (11)).

The behavior of *σ*^2^(*V*) is particularly interesting. In refs. ^21,33^, it is shown that (i) for liquids with a PEL that is Gaussian and harmonic, a minimum in *σ*^2^(*V*) implies that the liquid has a density anomaly (at densities where *dσ*^2^/*dV* > 0). Moreover, (ii) for a Gaussian and harmonic PEL, the minimum in *σ*^2^(*V*) implies that the liquid exhibits an LLCP^21^. Indeed, our results for quantum q-TIP4P/F water show a minimum in σ^2^ as well [see Supplementary Note 2 and Fig. S1 in the SM].

### Inherent Structures of q-TIP4P/F water sampled during vitrification at *P* = 0.1, 1000 MPa

In this section, we study briefly the properties of the IS sampled by q-TIP4P/F water upon vitrification (isobaric cooling) at *P* = 0.1 MPa and 1000 MPa. Since we are cooling to very low temperatures (*T* = 40 K), in this section we show results using *n*_*b*_ = 128 beads per ring polymers. Briefly, our PIMD simulations show that upon cooling, the system vitrifies into low-density amorphous ice (LDA) at *P* = 0.1 MPa, and HDA at *P* = 1000 MPa. In particular, we show that the ring polymers associated with the O/H atoms of q-TIP4P/F water increasingly expand with decreasing temperatures, but they collapse at the sampled IS.

**Fig. 8:**
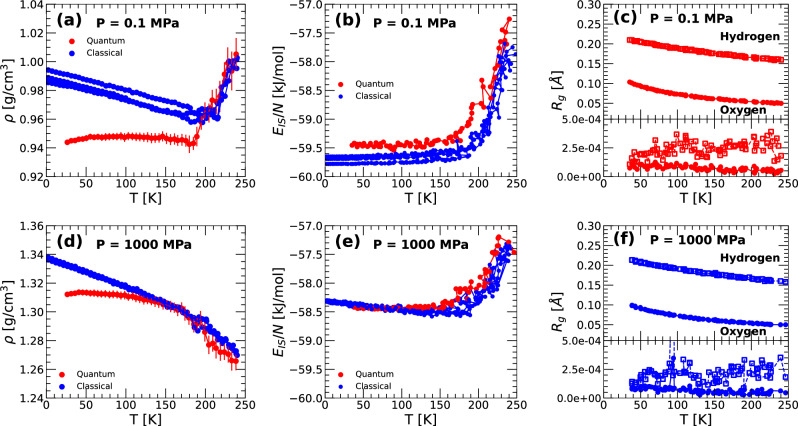
Thermodynamic and PEL properties of q-TIP4P/F water during vitrification (isobaric cooling) obtained from classical MD and PIMD simulations. **a** Density, **b** IS energy, and **c** radius of gyration of the ring-polymers associated with the O/H atoms of q-TIP4P/F water during the isobaric cooling at *P* = 0.1 MPa [cooling rate *q*_*T*_ = 10 K/ns]. **a**, **b**, **d**, **e** results obtained from PIMD and classical MD simulations are indicated by red and blue circles, respectively. **d**–**f** Same as **a**–**c** for q-TIP4P/F water isobarically cooled at *P* = 1000 MPa. Water enters the glass state (LDA and HDA) at the vitrification temperature *T*_*v*_ ≈ 150−180 K. In the glass state (*T* < *T*_*v*_), *ρ*(*T*) varies linearly (blue circles) or weakly with temperature (red circles); *E*_*IS*_(*T*) remains approximately constant. The *R*_*g*_(*T*) increases monotonically upon cooling, within the liquid and glass (LDA and HDA) states (upper panels of **c** and **f**). At the corresponding IS (lower panels of **c** and **f**), the ring polymers are collapsed, i.e., ${R}_{g}^{IS}(T)\approx 0$ at all temperatures considered.

Figure 8a, d show, respectively, the density of q-TIP4P/F water upon isobaric cooling at *P* = 0.1 MPa and *P* = 1000 MPa. In both cases, the density of the system *ρ*(*T*) shows a sudden change of slope during the cooling process, which signals, approximately, the temperature at which the liquid enters the glass state (at ≈ 150−180 K). To confirm this, we include in Fig. 8b, e the IS energy *E*_*IS*_(*T*) during the corresponding isobaric cooling process. As expected, below the vitrification temperature, *E*_*IS*_(*T*) is practically constant, as the amorphous ice (LDA or HDA) remains trapped in an IS of the PEL. A detailed description of the vitrification process of q-TIP4P/F water into LDA at *P* = 0.1 MPa, as observed in PIMD simulations, is included in ref. ^38^. Accordingly, we refer the reader to that work for additional details. The vitrification of q-TIP4P/F water into HDA at *P* = 1000 MPa is qualitatively similar to the vitrification of water at *P* = 0.1 MPa into LDA; see refs. ^41,43,44^ for the phenomenology associated with the vitrification of water into HDA upon cooling under pressure from computer simulations.

Figure 8c, f shows the radius of gyration of q-TIP4P/F water during the cooling PIMD simulations at (c) *P* = 0.1 MPa and (f) *P* = 1000 MPa. Upon cooling, the *R*_*g*_(*T*) of the O and H atoms of q-TIP4P/F water increases monotonically. This occurs while water is in the liquid and glass states (LDA and HDA). However, at the IS sampled during the cooling process, ${R}_{g}^{IS}(T)\approx 0$ at all temperatures studied, i.e., the ring-polymers associated with the O/H atoms are collapsed at the IS. This implies that the IS sampled in the LDA and HDA states of the quantum q-TIP4P/F water (PIMD simulations, RP-PEL) are also IS of the classical q-TIP4P/F water (MD simulations, CL-PEL). We note that the corresponding IS in the CL-PEL, sampled by the quantum and classical q-TIP4P/F water in the LDA and HDA states, seem to be remarkably similar. This follows from Fig. 8b, e, which shows that the values of *E*_*IS*_(*ρ*) sampled during the cooling process by the quantum and classical q-TIP4P/F water are very similar to one another.

**Fig. 9:**
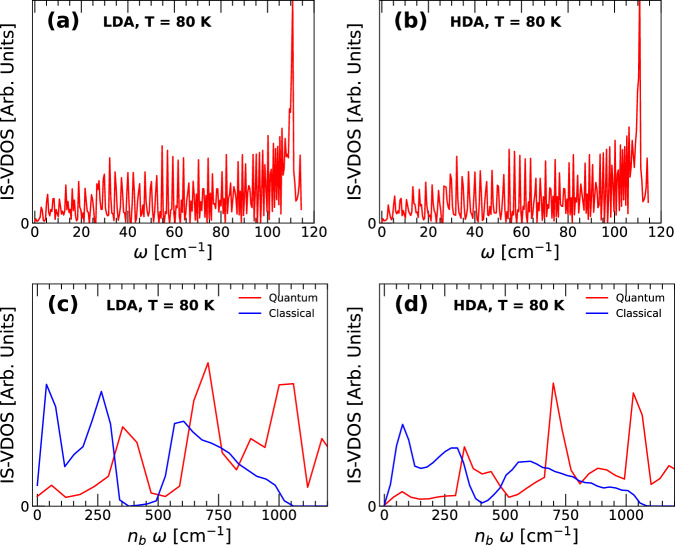
Vibrational density of states evaluated at the IS of the ring-polymer system associated to q-TIP4P/F water at *T* = 80 K. **a** LDA at *P* = 0.1 MPa (red line); **b** HDA at *P* = 1000 MPa (red line). **c**, **d** are magnifications of the IS-VDOS shown in **a**, **b**, respectively. Blue lines are the IS-VDOS of LDA and HDA obtained from classical MD simulations.

Figure 9 shows the IS-VDOS of the RP-PEL associated with LDA and HDA at *T* = 80 K. As for the case of liquid water (Fig. 3), the IS-VDOS of associated with both LDA and HDA extends to very large frequencies and is composed of large peaks over a large range of frequencies [Fig. 9a, b]. In particular, as shown in Fig. 9c, d, the IS-VDOS of LDA/HDA at low frequencies show no resemblance with the IS-VDOS of the corresponding classical LDA/HDA.

## Summary and discussion

There are only a few computational studies of the PEL of water available in the literature based on full-atomistic water models; specifically, the rigid SPC/E and TIP4P/2005 model^17,21^, and the flexible q-TIP4P/F model^18^. These studies are all based on *classical* MD simulations, where NQE is neglected. Instead, in this study, we employ PIMD simulations where NQE is inherently included. Since we also employ the q-TIP4P/F water model, our PIMD simulations include the effects of water flexibility and, hence, polarizability.

The PEL formalism was originally developed for the study of classical liquids and glasses. The extension of the PEL formalism to the case of liquids/glasses that obey quantum mechanics has been developed only recently^27,28^. Yet, these works have been limited to atomistic model liquids, and hence, the motivation of this work is to extend these ideas to the case of a molecular liquid, water. There are a few important differences in the PEL formalism when it is applied to classical and quantum liquids. Specifically, the PEL of a classical liquid is (a) uniquely defined by its potential energy as a function of the degrees of freedom in the system, and hence, (b) it is *T*-independent. Instead, the PEL associated with a quantum liquid is (a’) not uniquely defined, and (b’) it is *T*-dependent. It is *T*-dependent because the spring constants associated with the ring-polymers representing the atoms of the liquid vary with temperature. The PEL of a quantum liquid is not uniquely defined because it depends on the number of beads per ring-polymer used in the path-integral calculations (e.g. PIMD simulations). Yet, for a fixed value of *n*_*b*_, for which all thermodynamic properties of interest converge, a PEL for the corresponding quantum liquid can be defined and the PEL formalism can be applied^27^. In PIMD simulations, and hence in our extension of the PEL formalism, one must use a finite value of *n*_*b*_ that is large enough so that the relevant thermodynamic properties of the system are well-converged (i.e., these properties do not change upon further increase in *n*_*b*_)^25^. In this regard, a natural question follows, how do the different properties of the RP-PEL vary as *n*_*b*_ increases? We are currently addressing this question, and the results will be presented in a separate work. The PEL formalism for quantum liquids developed here is exact, i.e., it is based on the true water model considered and it is based on the path-integral formalism of statistical mechanics. It is also possible to apply the PEL formalism to quantum liquids using effective interactions, such as the case of centroid molecular dynamics (CMD)^45,46^. It would be interesting in the future to apply the PEL formalism to study quantum liquids using effective force fields, including CMD, since such techniques would reduce the computational cost associated with the PEL formalism, particularly for systems at low temperatures.

Our computer simulations show that the PEL associated with q-TIP4P/F water, for a fixed number of beads per ring-polymer *n*_*b*_ = 32, is Gaussian (i.e., *E*_*IS*_(*T*) obeys Eq. (16)) for all densities considered (0.80 ≤ *ρ* ≤ 1.40 g/ cm^3^) and for *T* ≤ 280 K (Fig. 7a). However, we find that the harmonic approximation of the PEL does not hold for the case of q-TIP4P/F water (Fig. 6a). This should not be surprising since the harmonic approximation does not hold for the PEL of *classical* rigid/flexible water models^17,18,21^. It follows that the basins in the PEL of water, based on quantum and classical water models, contain significant anharmonicities that need to be taken into account when applying the PEL formalism (see Fig. 6b).

Two very important, and subtle, results follow from our PIMD simulations. Consistent with refs. ^27,28^, (i) our PIMD simulations show that the ring-polymers representing the O/H atoms of q-TIP4P/F water are all collapsed at the IS [after minimization of the potential energy of the ring-polymer system (Fig. 2)]. This finding allows one to calculate the IS vibrational normal mode frequencies of the ring-polymer system associated to q-TIP4P/F water (accessible from PIMD simulations), from the corresponding vibrational normal mode frequencies of the classical q-TIP4P/F water system (accessible from MD simulations)^28^. Specifically, we confirm that Eq. (20) provides the IS vibrational normal mode frequencies of the ring-polymer system (at no extra computational cost) from the normal mode frequencies of the corresponding classical system. The direct calculation of such frequencies, which involves the diagonalization of the *mass-weighted* Hessian matrix of the ring-polymer/water system, is computationally very expensive for even small system sizes (*N* ≈ 1000, *n*_*B*_ ≈ 30). Eq. (20) simplifies enormously the application of the PEL formalism to quantum liquids. For example, the vibrational density of states and shape function, which is a very important PEL property to describe a liquid, can be calculated in a straightforward manner (Figs. 3 and 4). Interestingly, we find that the collapse of the ring-polymers representing the O/H atoms of q-TIP4P/F water is not limited to the liquid state (~*T* > 200 K). As shown in the section ‘Inherent Structures of q-TIP4P/F Water Sampled During Vitrification at *P* = 0.1, 1000 MPa’, the O/H ring-polymers collapse even at temperatures as low as *T* = 40 K, and at low and high pressures (*P* = 0.1, 1000 MPa), at conditions where water is in the glassy (LDA and HDA) states.

The collapse of the ring-polymers associated with the O/H atoms of q-TIP4P/F water at the IS sampled by the system implies that the IS of the ring-polymer/quantum water, in the ring-polymer PEL (RP-PEL), are also IS of the classical q-TIP4P/F water system, in the classical PEL (CL-PEL); see refs. ^27,28^. Consistent with this finding, our PIMD simulations show that the IS properties of the quantum and classical q-TIP4P/F water [*E*_*IS*_(*T*) in Fig. 1b, and the PEL variables *E*_0_(*V*) and *σ*^2^(*V*) in Fig. 7b, c] are very close to one another. Similarly, the structural properties of q-TIP4P/F water at the IS obtained from classical and PIMD simulations practically overlap with one another (see Supplementary Note 3, 6 and Figs. S6–S8 in the SM). Instead, the vibrational properties of the ring-polymer system and classical q-TIP4P/F water (*E*_*vib*_(*T*) in Fig. 1b, and ${{\mathcal{S}}}(T)$ in Fig. 4) are fundamentally different. This is not surprising since the CL-PEL of q-TIP4P/F water is a hypersurface in (9*N* + 1)-dimensional space, while the PEL of the quantum q-TIP4P/F water (RP-PEL) is a hypersurface in a (9*Nn*_*b*_ + 1)-dimensional space. We stress that, at the molecular level, the q-TIP4P/F water molecules in MD and PIMD simulations are rather different. In addition to the non-negligible delocalization of the H atoms, the inclusion of NQE affects the average distance between hydrogen-bonded water molecules, $\langle {d}_{OO}^{HB} \rangle$, and the linearity of the hydrogen-bonds (i.e., the HOO angle $\langle {\theta }_{HOO}^{HB} \rangle$ between molecules forming a hydrogen-bond). A detailed analysis of the structure of q-TIP4P/F water is included in Supplementary Note 6 in the SM. For example, we find that, at all densities studied, the inclusion of NQE leads to a softer hydrogen-bond network and a less structured q-TIP4P/F water, with slightly longer $\langle {d}_{OO}^{HB} \rangle$ and larger $\langle {\theta }_{HOO}^{HB} \rangle$.

One of the aims of our work was (i) to test the PEL formalism for the case of water, a realistic and molecular liquid, (ii) when the system evolves according to the laws of quantum mechanics. From a fundamental point of view, extending the PEL formalism to quantum liquids allows one to explain the behavior of liquids and glasses with a unique theoretical framework, that is, independent of the laws (classical or quantum mechanics) that control the evolution of the system of interest. From a practical point of view, extending the PEL formalism to quantum liquids may allow one to provide a thermodynamic description (e.g., an expression for the free energy of the system) in terms of topographic properties of the PEL (accessible in computer simulations). At present, this has been done only for classical liquids^11–13,15,17,18,21,24^. In the case of water, the PEL formalism has been used to locate quite successfully the location of the LLCP of *classical* models at conditions where computer simulations are challenging^17,18,21,24^. It would be important in the future to test whether the PEL formalism for q-TIP4P/F water, in the presence of NQE, can reproduce successfully the equation-of-state as obtained in PIMD simulations as well as the location of the associated LLCP. It would also be interesting to apply the PEL formalism to more advanced water models, including MB-pol^47–49^ and machine-learning models based on DFT calculations^50,51^. We expect that, qualitatively, the results based on q-TIP4P/F water model will hold for other water models. After all, computational studies applying the PEL formalism to different *classical* water and water-like models provide qualitatively similar results^17,18,20–22,24,52^.

The PEL formalism, extended to quantum liquids, allows one to explore the isotope effects in water where such effects are particularly important^53–55^. A few natural questions follow, e.g., what are the features of the PEL that distinguish H_2_O from its isotopes D_2_O and T_2_O? and, in particular, is it possible to predict the phase behavior of D_2_O and T_2_O by using information based solely on the PEL of H_2_O? There are theoretical models indicating that simple scaling rules can be used to predict the thermodynamic properties of D_2_O based solely on the corresponding properties of H_2_O^56–58^. We expect that the main differences in the PEL of H_2_O and its isotopes are related to the “shape" (curvature) of the corresponding RP-PEL basins (about the IS). These and other questions related to isotope effects in water will be addressed in separate studies.

## Methods

Our results are based on path-integral molecular dynamics (PIMD) simulations of a system composed of *N* = 512 water molecules in a cubic box with periodic boundary conditions. Water molecules are represented using the flexible q-TIP4P/F water model introduced in ref. ^29^. The q-TIP4P/F water model is based on the rigid TIP4P/2005 model for water^59^, which is commonly used in classical MD simulations^17,20,60,61^. The q-TIP4P/F water model was optimized for PIMD simulations but it has been used in MD simulations as well^18,29,31,32,41,62^. This water model reproduces remarkably well the properties of liquid water^29,31,32^, ice *I*_*h*_^30,38,63,64^, and LDA^38,41^ at *P* = 0.1 MPa. The q-TIP4P/F model incorporates intramolecular flexibility by modeling the OH-covalent bond potential energy with a fourth-order polynomial expansion of a Morse potential and a harmonic potential to model the potential energy of the HOH angle.

Here, we perform PIMD simulations at constant (*N*, *V*, *T*) over a wide range of temperatures, 180 ≤ *T* ≤ 400 K, and densities, 0.80 ≤ *ρ* ≤ 1.40 g/cm^3^. The temperature is controlled using the stochastic (local) path-integral Langevin equation thermostat^65^ with the thermostat collision frequency parameter *γ* = 0.1 ps^−1^. In the PIMD simulations, the time step is *dt* = 0.25 fs and the number of beads per ring-polymer/atom is set to *n*_*b*_ = 32; as shown in refs. ^31,32^, this value of *n*_*b*_ is large enough to obtain converged values for the thermodynamic and structural properties of q-TIP4P/F water in the liquid state. Short-range (Lennard-Jones pair potential) interactions are calculated using a cutoff of *r*_*c*_ = 1.0 nm, and the long-range electrostatic interactions are computed using the reaction field technique^66^ with the same cutoff *r*_*c*_. In the reaction field technique, the dielectric constant (relative permittivity) of the continuum medium beyond the cutoff radius *r*_*c*_ is set to 78.3. All of our PIMD simulations are performed using the OpenMM software package (version 7.4.0)^67^.

PIMD simulations are performed for a time interval *t*_*tot*_ = *t*_*eq*_ + *t*_*prod*_ where *t*_*eq*_ is the equilibration time and *t*_*prod*_ is the production time, during which data analysis is performed. The values of *t*_*tot*_, *t*_*eq*_, and *t*_*prod*_ depend on the state point simulated. The total PIMD simulation time is in the range *t*_*tot*_ = 5–200 ns, depending on the *T* and *V* considered. To confirm that the system reaches equilibrium in a given simulation, we calculate the mean-square displacement (MSD) of the water molecules as a function of time. In all cases, we confirm that the PIMD simulations satisfy the requirement that *t*_*eq*_, *t*_*prod*_ > *τ*, where *τ* is the time that it takes for the MSD of the water molecules to reach a value of 1.0 nm^2^. Note that an MSD of 1.0 nm^2^ roughly indicates that molecules diffuse, in average, ≈ 0.3 nm, which is approximately the OO distance between neighboring water molecules. We also perform classical MD simulations of q-TIP4P/F water by setting *n*_*b*_ = 1 in the PIMD simulations. The time step for the MD simulations is *dt* = 0.5 fs (see ref. ^18^ for details).

### IS analysis

During the MD/PIMD simulations, we save a total of 25 equally spaced configurations for each state point simulated. For each of these configurations, we minimize the potential energy of the system using the L-BFGS-B algorithm^68^ and obtain the corresponding local minima (IS) of the PEL. When unclear, we will refer as CL-PEL to the PEL of the classical q-TIP4P water (MD simulations); the PEL of the ring-polymer system associated with quantum q-TIP4P water (PIMD simulations) will be denoted as RP-PEL. The energy of the IS, *E*_*IS*_, is obtained directly from the minimization algorithm. In order to calculate the curvature of the CL-PEL basins about the corresponding IS, we calculate the *mass-weighted* Hessian matrix (at the IS) using analytical expressions for the matrix components, based on the q-TIP4P/F potential energy function. The *mass-weighted* Hessian matrix is then diagonalized numerically to calculate the corresponding eigenvalues, which provide the normal mode frequencies of the classical q-TIP4P/F water. To calculate the curvature of the RP-PEL basins about the corresponding IS, we employ the same method used in ref. ^28^; see also section “Vibrational Density of States of the Ring-Polymer System at the IS”.

## Supplementary information

Supplementary Material

## Data Availability

The authors confirm that the data supporting the findings of this study are available within the article and its SM. In the SM, we derive the equation for the eigenvalues of the mass-weighted Hessian matrix of the ring-polymer system associated with (quantum) q-TIP4P/F water [Eq. 20]. Also included are additional results from PIMD simulations, (i) where we show the phase diagram of q-TIP4P/F water (using the reaction field technique to treat electrostatic interactions), (ii) validate Eq. (20) for different cluster sizes of water, (iii) provide additional details on the calculation of the shape function, and (iv) discuss the structural properties (RDFs, hydrogen bonding, and local order metrics) of q-TIP4P/F water from MD and PIMD simulations at the instantaneous and IS configuration.
