# Supplementary material for: Potential energy landscape formalism for quantum molecular liquids
Source: Commun Chem. 2024 Dec 4;7:289. doi: 10.1038/s42004-024-01342-9 (PMC11618503; doi:10.1038/s42004-024-01342-9)
Supplement: Supplementary file 1 — Supplementary Material [file 42004_2024_1342_MOESM1_ESM.pdf]

Supplementary Material for 'Potential Energy Landscape Formalism for Quantum Molecular Liquids'

Ali Eltareb[1,2,*], Yang Zhou[1,2,*], Gustavo E. Lopez[3,4,*], and Nicolas Giovambattista[1,2,4*]

[1] *Department of Physics, Brooklyn College of the City University of New York, Brooklyn, New York 11210, United States*

[2] *Ph.D. Program in Physics, The Graduate Center of the City University of New York, New York, NY 10016, United States*

[3] *Department of Chemistry, Lehman College of the City University of New York, Bronx, New York 10468, United States*

[4] *Ph.D. Program in Chemistry, The Graduate Center of the City University of New York, New York, NY 10016, United States*

[*] aeltareb@gradcenter.cuny.edu, yzhou4@gradcenter.cuny.edu, gustavo.lopez1@lehman.cuny.edu, ngiovambattista@brooklyn.cuny.edu

I. SUPPLEMENTARY NOTE 1

Normal Modes Frequencies of the Ring-Polymer System at the Inherent Structures

In this section, we calculate the eigenvalues of the *mass-weighted* Hessian matrix of the ring-polymer system associated to (quantum) q-TIP4P/F water. We consider the case where (i) the ring-polymer system is at an IS of the RP-PEL, and (ii) the ring-polymers are collapsed. The discussion below is built upon the calculations in the Appendix of Ref. [1]. In Ref. [1], we considered a quantum monatomic liquid composed of N atoms represented by ring-polymers composed of n_b beads and with a Hamiltonian given by,

$$\mathcal{H}_{RP}(\boldsymbol{R},\boldsymbol{P}) = \sum_{i=1}^{N}\sum_{k=1}^{n_b}\frac{(\boldsymbol{p}_i^k)^2}{2m_i'} + \sum_{i=1}^{N}\sum_{k=1}^{n_b}\frac{1}{2}k_{sp}(\boldsymbol{r}_i^{k+1}-\boldsymbol{r}_i^k)^2 + \frac{1}{n_b}\sum_{k=1}^{n_b}U(\boldsymbol{r}_1^k,\boldsymbol{r}_2^k,...,\boldsymbol{r}_N^k) \quad (1)$$

where $k_{sp} = m_i n_b/(\beta\hbar)^2$ is the ring-polymers bead mass and $m_i' = n_b m_i$; atoms have identical mass, i.e., $m_i = m$ for all i. The potential energy of the ring-polymer/atoms system is then,

$$\mathcal{U}_{RP}(\boldsymbol{R}) = \sum_{i=1}^{N}\sum_{k=1}^{n_b}\frac{1}{2}k_{sp}(\boldsymbol{r}_i^{k+1}-\boldsymbol{r}_i^k)^2 + \frac{1}{n_b}\sum_{k=1}^{n_b}U(\boldsymbol{r}_1^k,\boldsymbol{r}_2^k,...,\boldsymbol{r}_N^k) \quad (2)$$

Eqs. 1 and 2 are identical to Eqs. 4 and 5 in the main manuscript with $n \to N$ and $k_i^{sp} \to k_{sp}$. The Hessian matrix of the ring-polymer system defined by Eqs. 1 and 2 is a $(3Nn_b \times 3Nn_b)$-square matrix with components given by

$$[\boldsymbol{H}^{RP}]_{i,k,\alpha}^{j,l,\beta} = \frac{\partial^2\mathcal{U}_{RP}}{\partial r_{i,\alpha}^k \partial r_{j,\beta}^l} \quad (3)$$

where (i,k,α) define the location of the matrix element within a row, and (j,l,β) define the location of the matrix element within a column. As discussed in detail in Ref. [1], $i,j = 1,2,..,N$; $k,l = 1,2,..,n_b$; $\alpha,\beta \in \{x,y,z\}$. In the appendix of Ref. [1], it is shown that the $3Nn_b$ eigenvalues of the Hessian matrix defined in Eq. 3 are given by

$$\omega_{i,j}^2 = \frac{\omega_{i,0}^2}{n_b} - 2k_{sp}\left[\cos\left(\frac{2\pi}{n_b}j\right) - 1\right] \quad (4)$$

where $i = 1,2,..,3N$ and $j = 1,2,..,n_b$. In the expression above, $\{\omega_{i,0}^2\}$ are the eigenvalues of the Hessian matrix of the *classical* version of the monatomic liquid considered (see Ref. [1]).

Strictly speaking, the normal mode frequencies of the ring-polymer system associated to the quantum liquid are given by the eigenvalues of the corresponding *mass-weighted* Hessian

matrix,

$$[\boldsymbol{H}^{RP}]_{i,k,\alpha}^{j,l,\beta} = \frac{1}{\sqrt{m'^k_i m'^l_j}} \frac{\partial^2 \mathcal{U}_{RP}}{\partial r^k_{i,\alpha} \partial r^l_{j,\beta}} \tag{5}$$

where m'^k_i is the mass of bead k belonging to ring-polymer i. We note, however, that $m'^k_i \to m'_i = n_b m_i$ since all beads belonging to a given ring-polymer have the same mass. In the case of monatomic atomistic systems, one usually uses reduced units where $m_i = m = 1$. This is not the case for mixtures, such as silica [2, 3], and molecular systems, such as q-TIP4P/F water [4]. Next, we obtain the eigenvalues of the *mass-weighted* Hessian matrix defined in Eq. 5 for the case of the ring-polymer system associated to (quantum) q-TIP4P/F water.

By following the same calculations of the Appendix in Ref. [1], one can easily show that for the case of q-TIP4P/F water, the matrix elements defined in Eq. 5 are given by

$$[\boldsymbol{H}^{RP}]_{i,k,\alpha}^{j,l,\beta} = \frac{1}{n_b\sqrt{m_i m_j}} k^i_{sp}(2\delta^{j,l,\beta}_{i,k,\alpha} - \delta^{j,l,\beta}_{i,k+1,\alpha} - \delta^{j,l,\beta}_{i,k-1,\alpha}) + \frac{1}{\sqrt{m_i m_j}} \frac{\delta^l_k}{n_b^2} \frac{\partial^2 U_{q-TIP4P/F}}{\partial r^k_{i,\alpha} \partial r^l_{j,\beta}} \tag{6}$$

where $U_{q-TIP4P/F} = U(\boldsymbol{r}^k_1, \boldsymbol{r}^k_2, ..., \boldsymbol{r}^k_{9N})$ is the potential energy function of the *classical* q-TIP4P/F water evaluated at the given IS. $\delta^{j,l,\beta}_{i,k,\alpha} = 1$ if $i = j$ and $k = l$ and $\alpha = \beta$; $\delta^{j,l,\beta}_{i,k,\alpha} = 0$, otherwise (for q-TIP4P/F water, $i, j = 1, 2, .., 9N$; $k, l = 1, 2, .., n_b$; $\alpha, \beta \in \{x, y, z\}$). The last term of Eq. 6 is the *mass-weighted* Hessian matrix of the $9N$-atoms system evaluated at the IS of the CL-PEL, i.e.,

$$[\boldsymbol{H}^{CL}]_{i,\alpha}^{j,\beta} = \frac{1}{\sqrt{m_i m_j}} \frac{\partial^2 U_{q-TIP4P/F}}{\partial r^k_{i,\alpha} \partial r^l_{j,\beta}} \delta^l_k \tag{7}$$

It follows that the *mass-weighted* Hessian of the ring-polymer system at the given IS can be written as the sum of a term that depends solely on the ring-polymer springs, and the *mass-weighted* Hessian matrix of the classical water system, i.e.,

$$[\boldsymbol{H}^{RP}]_{i,k,\alpha}^{j,l,\beta} = \frac{1}{n_b\sqrt{m_i m_j}} k^i_{sp}(2\delta^l_k - \delta^l_{k+1} - \delta^l_{k-1})\delta^{j,\beta}_{i,\alpha} + \frac{\delta^l_k}{n_b^2} [\boldsymbol{H}^{CL}]^{j,\beta}_{i,\alpha} \tag{8}$$

The *mass-weighted* Hessian matrix of the ring-polymer system can be written as (see

Ref. [1])

$$\boldsymbol{H}^{RP} = -\frac{1}{(\hbar\beta)^2}\begin{pmatrix} (-2)\mathbf{1} & \mathbf{1} & & & \mathbf{1} \\ \mathbf{1} & (-2)\mathbf{1} & \mathbf{1} & & \\ & \mathbf{1} & \ddots & \ddots & \\ & & \ddots & (-2)\mathbf{1} & \mathbf{1} \\ \mathbf{1} & & & \mathbf{1} & (-2)\mathbf{1} \end{pmatrix} + \frac{1}{n_b^2}\begin{pmatrix} \boldsymbol{H}^{CL}(\boldsymbol{R}_{IS}) & & & & \\ & \ddots & & & \\ & & \boldsymbol{H}^{CL}(\boldsymbol{R}_{IS}) & & \\ & & & \ddots & \\ & & & & \boldsymbol{H}^{CL}(\boldsymbol{R}_{IS}) \end{pmatrix} \quad (9)$$

where $\mathbf{1}$ is the $(9N \times 9N)$-identity square matrix; $\boldsymbol{H}^{CL}(\boldsymbol{R}_{IS})$ is the $(9N \times 9N)$-Hessian matrix of classical water evaluated at the IS $\boldsymbol{R}_{IS}$. Eq. 9 is identical to Eq. 35 in the Appendix of Ref. [1] after (i) replacing $k_{sp} \to 1/(\beta\hbar)^2$, and (ii) where $\boldsymbol{H}^{CL}(\boldsymbol{R}_{IS})$ is now the *mass-weighted* Hessian of the classical system (q-TIP4P/F water). Accordingly, following the same mathematical steps as in Ref. [1], one can show that

$$\omega_{i,j}^2 = \frac{\omega_{i,0}^2}{n_b^2} - \frac{2}{(\hbar\beta)^2}\left[\cos\left(\frac{2\pi}{n_b}j\right) - 1\right] \quad (10)$$

where $i = 1, 2, ..., 9N$ and $j = 1, 2, ..., n_b$. In the expression above, $\{\omega_{i,0}^2\}$ are the eigenvalues (normal mode frequencies squared) of the *mass-weighted* Hessian matrix of the *classical* version of q-TIP4P/F water.

We note that in Ref. [1], it is stated that the normal mode frequencies at the IS of the RP-PEL are given by Eq. 4. Eq. 4 correctly gives the eigenvalues of the Hessian matrix (Eq. 3). However, as we state here, the normal mode frequencies at the IS of the RP-PEL are the eigenvalues of the *mass-weighted* Hessian matrix (Eq. 5). Accordingly, the true normal mode vibrational frequencies at the IS of the RP-PEL are given by Eq. 10; Eq. 10 provides the correct frequencies $\{\omega_{i,j}\}$ that define the shape function in Eq. 14 of the main manuscript. The effect of using Eq. 4 instead of Eq. 10, is to re-scale (incorrectly) the frequencies $\{\omega_{i,j}\}$ by $\sqrt{n_b}$ [since the atoms mass in Ref. [1] is $m = 1$ (reduced units)]. This re-scaling implies that some results of Ref. [1] need to be corrected. Specifically, the extra pre-factor $\sqrt{n_b}$ included in the frequencies $\{\omega_{i,j}\}$ reported in Ref. [1] implies that the values of $a(T)$ and

$\mathcal{S}(T)$ reported in Figs. 5, 6, and 7a of that work are (incorrectly) shifted by a constant. Yet, the conclusions of Ref. [1] remain unaffected.

II. SUPPLEMENTARY NOTE 2

The Liquid-Liquid Critical Point of q-TIP4P/F water

Previous classical molecular dynamics (MD) and path-integral MD (PIMD) simulations of q/TIP4P/F water, with electrostatic interactions evaluated using the Particle-Mesh Ewald sum technique, show that this water model exhibits a liquid-liquid critical point (LLCP) [5]; see Table S1. Sensitivity of the LLCP location to the method employed to treat the electrostatic interactions are expected; see, e.g., Ref. [6]. Indeed, classical MD simulations of q-TIP4P/F water with electrostatic interactions evaluated using the reaction field technique shift slightly the location of the LLCP [4]; see Table S1. Our results from PIMD simulations using the reaction field technique indicate that the LLCP is located at ($\rho_c = 1.03$ g/cm^3, $T_c = 180$ K, $P_c = 135$ MPa). To show this, we include in Fig. S1(a) the $P-T$ phase diagram of q-TIP4P/F water obtained from PIMD simulations using the reaction-field technique to treat the electrostatic interactions. Included in Fig. S1(a) are the corresponding results from classical MD simulations (open squares) reported in Ref. [4]. In both cases, and consistent with thermodynamic relationships [7], the isochores for volumes close to the LLCP volume intersect at the LLCP (red and blue stars). At $T < T_c$, q-TIP4P/F water can exist in two distinct liquid phases, low-density and high-density liquid (LDL and HDL). The LLCP for quantum q-TIP4P/F water (red star) is located at a slightly lower temperature and pressure than the LLCP for classical q-TIP4P/F water (blue star) [4]. Overall the phase diagrams shown in Fig. S1(a) are fully consistent with Refs. [5, 8–10] where it is shown that introducing NQE shifts the LLCP towards lower temperatures and pressures.

Fig. S1(b) shows the pressure of q-TIP4P/F water as function of volume from PIMD simulations at selected temperatures. The presence of a van der Waals loop at $T < T_c \approx 190$ K confirms the existence of a liquid-liquid phase transition (LLPT) in q-TIP4P/F water. In addition, we find that the total energy of the system along isotherms, $E(T)$, exhibits a concave region [where $(\partial^2 E/\partial V^2)_{N,T} < 0$], which is consistent with the existence of a LLPT at low temperatures (see, e.g., Ref. [5]).

	Reaction Field	Particle Mesh Ewald Sum
Quantum	$\rho_c \approx 1.03$ g/cm^3, $T_c \approx 180$ K, $P_c \approx 135$ MPa	$\rho_c = 1.02$ g/cm^3, $T_c = 159$ K, $P_c = 167$ MPa
Classical	$\rho_c = 1.04$ g/cm^3, $T_c = 190$ K, $P_c = 150$ MPa	$\rho_c = 1.03$ g/cm^3, $T_c = 175$ K, $P_c = 203$ MPa

TABLE S1: The location of the LLCP of q-TIP4P/F water obtained from PIMD and Classical MD simulations where the electrostatic interactions are evaluated using either the Particle-Mesh Ewald (PME) sum (from Ref. [5]) or the reaction field technique (from Ref. [4]). Note that the location of the LLCP using the PME sum was *estimated* using the Two-State-Equation of State [5, 11–14].

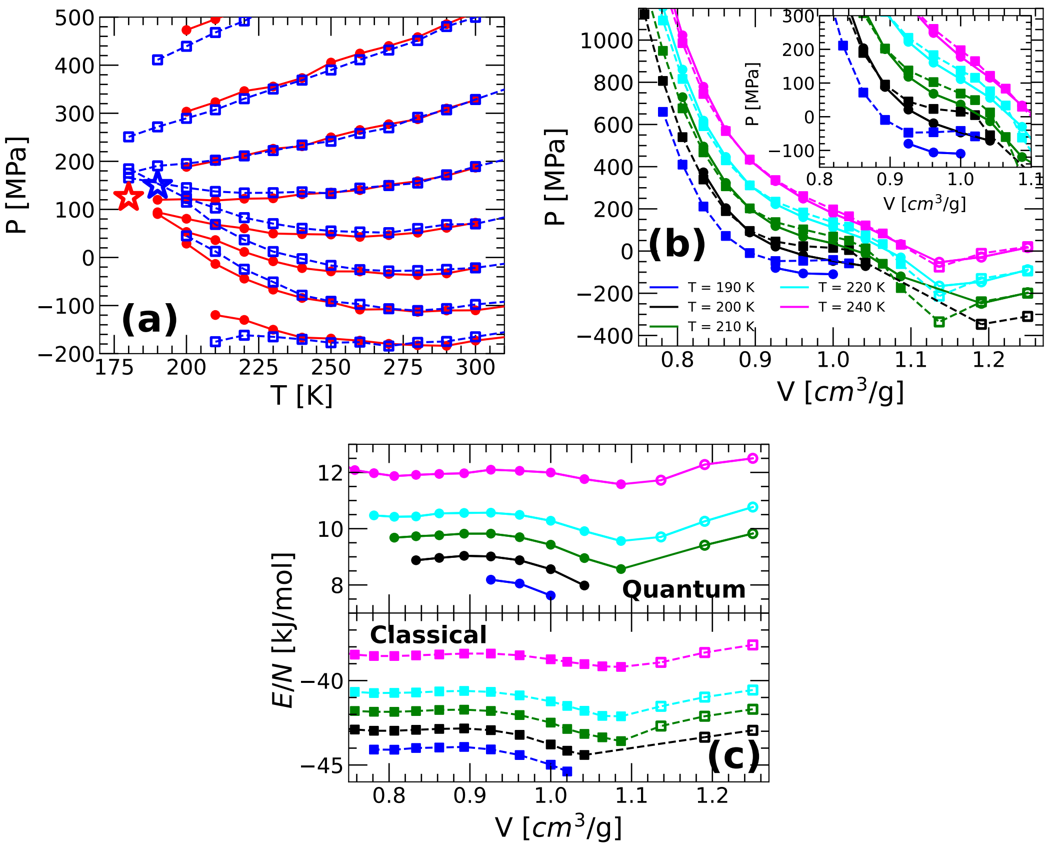

FIG. S1. (a) Pressure as a function of temperature of q-TIP4P/F water along selected isochores. Blue and red lines are results from classical MD and PIMD simulations, respectively. In both cases, isochores intersect at the corresponding LLCP (red and blue stars). Including NQE shifts the location of the LLCP towards slightly lower temperatures and pressures. Lines correspond to densities (bottom-to-top) $\rho = 0.92,\ 1.20$ g/cm^3 in steps of 0.04 g/cm^3. (b) Pressure and (c) total energy of q-TIP4P/F water as a function of volume at selected temperatures; results are from PIMD (circles) and MD simulations (squares). Consistent with the presence of a LLCP, the $P(V)$ of classical q-TIP4P/F water exhibits an inflection point at $T = T_c \approx 190$ K. Similarly, $P(V)$ exhibits a van der Waals loop at $T < Tc$ while $E(V)$ has a concave region where $\left(\partial^2 E/\partial V^2\right)_{N,T} < 0$. In (b) and (c), solid and empty symbols indicate the liquid and vapor states.

III. SUPPLEMENTARY NOTE 3

Inherent structure Pressure of q-TIP4P/F water

Fig. S2(a) shows the IS pressure of q-TIP4P/F water, $P_{IS}(T)$, as a function of temperature (solid circles). We define $P_{IS}(T)$ as the value given by the pressure virial expression evaluated at the IS. We note that the difference in the $P_{IS}(T)$ values obtained from MD/PIMD simulations are < 100 MPa, comparable to the differences in the corresponding values of $P(T)$ shown in Fig. S1(a).

We also include in Fig. S2(b) the vibrational pressure of q-TIP4P/F water, $P_{vib}(T) \equiv P(T) - P_{IS}(T)$ obtained from classical MD (dashed lines) and PIMD simulations (solid lines).

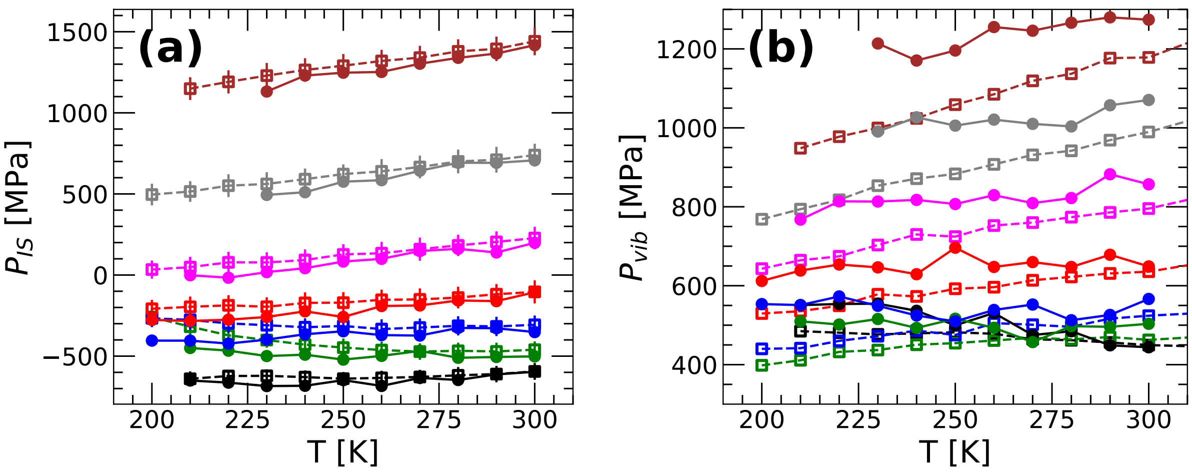

FIG. S2. (a) Inherent structure pressure of q-TIP4P/F water obtained from PIMD simulations (solid lines) and classical MD simulations (dashed lines). Densities are $\rho = 0.92 - 1.40$ g/cm^3 in steps of 0.08 g/cm^3, from bottom-to-top. (b) Vibrational pressure $P_{vib}(T) \equiv P(T) - P_{IS}(T)$ of q-TIP4P/F water as a function of temperature obtained from (a) and Fig. S1(a).

IV. SUPPLEMENTARY NOTE 4

Vibrational Density of States at the Inherent Structures for Water Clusters

In this section, we show that the IS normal mode frequencies of the ring-polymer system associated to q-TIP4P/F water, obtained from PIMD simulations, are in full agreement with Eq. 20 of the main manuscript, i.e., they are given by

$$\omega_{i,j}^2 = \frac{\omega_{i,0}^2}{n_b^2} - \frac{2}{(\hbar\beta)^2}\left[\cos\left(\frac{2\pi}{n_b}j\right) - 1\right] \tag{11}$$

where $i = 1, 2, ..., 9N$ and $j = 1, 2, ...n_b$. The set $\{\omega_{i,j}^2\}$ are the $(9Nn_b)$ eigenvalues of the *mass-weighted* Hessian matrix (normal mode frequencies) of the ring-polymer system associated to the quantum q-TIP4P/F water. The set $\{\omega_{i,0}^2\}_{i=1,2...9N}$ are the $9N$ eigenvalues of the *mass-weighted* Hessian matrix of the classical q-TIP4P/F water (e.g., calculated from classical MD simulations). As explained in the main manuscript, diagonalization of the *mass-weighted* Hessian matrix is computational expensive for the case of q-TIP4P/F water, even for our small system composed of $N = 512$ molecules and using $n_b = 32$ beads per ring-polymer. Accordingly, here, we validate Eq. 11 for the case of small clusters of q-TIP4P/F water molecules.

We perform PIMD simulations of clusters composed of $N = 4,\ 8,\ 16,\ 32,\ 64,\ 96$ q-TIP4P/F water molecules at $T = 200$ K. In these simulations, the water cluster is placed in a large cubic box (side length of 10 nm) and the simulation is performed for 500 ps. For each run, we save six configurations separated by 50 ps and the corresponding IS and the *mass-weighted* Hessian matrix are then calculated. Fig. S3 shows the vibrational density of states of the q-TIP4P/F water clusters evaluated at the IS (IS-VDOS). Blue lines are the IS-VDOS calculated numerically, based on the PIMD simulations. The red lines are the IS-VDOS obtained by using Eq. 11 with the frequencies $\{\omega_{i,0}\}_{i=1,2...9N}$ calculated independently from classical MD simulations of the q-TIP4P/F water clusters studied. The overlap among the blue and red lines in all the panels of Fig. S3 validates the use of Eq. 11 to calculate the IS-VDOS of q-TIP4P/F water for systems of any size.

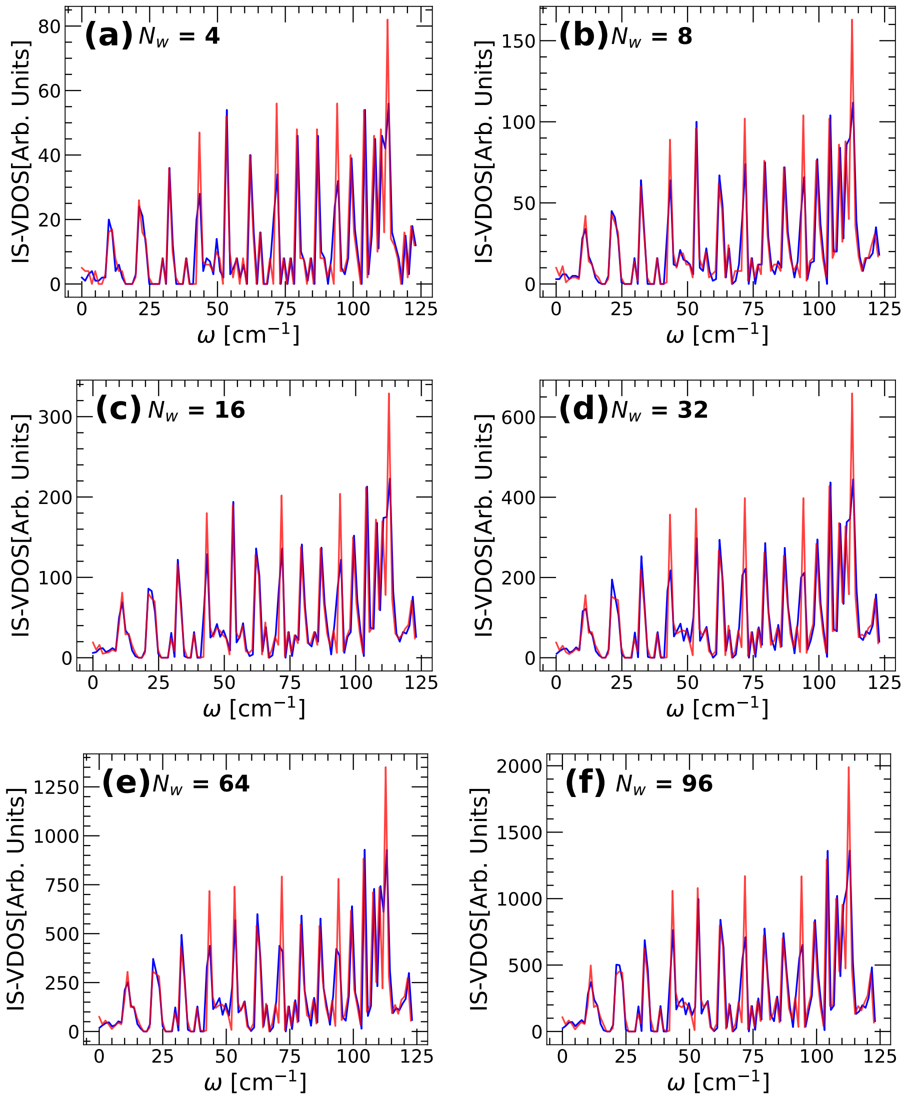

FIG. S3. Vibrational density of states of q-TIP4P/F water clusters evaluated at the corresponding inherent structures (IS-VDOS) at T = 200 K. Results are from PIMD simulations of clusters composed of (a) N = 4, (b) N = 8, (c) N = 16, (d) N = 32, (e) N = 64, (f) N = 96 water molecules. Blue lines are the IS-VDOS obtained by calculating numerically the Hessian matrix of the water clusters (evaluated at the IS); red lines are the IS-VDOS predicted by Eq. 11.

A snapshot of the cluster composed of $N = 96$ q-TIP4P/F water molecules is included in Fig. S4(a); Fig. S4(b) shows the snapshot of the system at the corresponding IS. Consistent with the discussion in the main manuscript, the ring-polymers associated to the O/H atoms of the water molecules collapse at the IS.

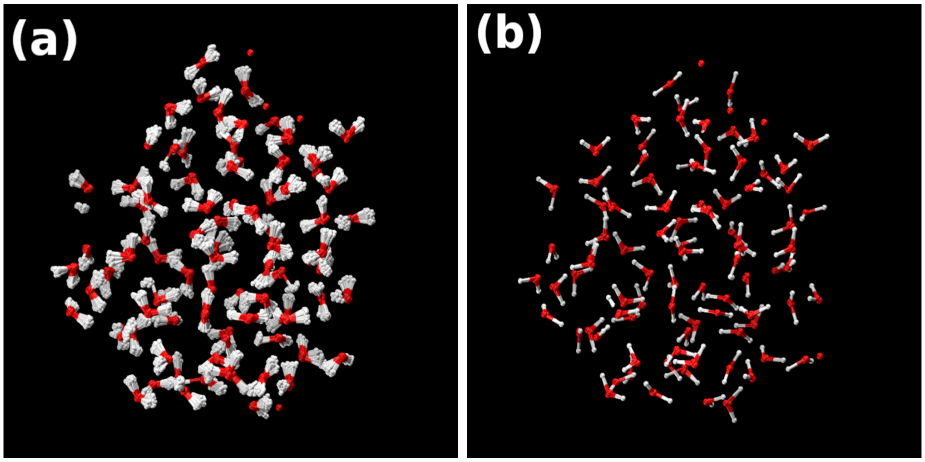

FIG. S4. (a) Snapshot of a cluster composed of $N = 96$ q-TIP4P/F water molecules at $T = 200$ K showing that the O and H atoms are delocalized. (b) Snapshot of the water cluster at the IS corresponding to the configuration shown in (a). At the IS, the ring-polymers associated to the O/H atoms of the water molecules are collapsed.

V. SUPPLEMENTARY NOTE 5

Shape Function $\mathcal{S}$ of q-TIP4P/F Water

In the main manuscript, we state that the shape function $\mathcal{S}(N, V, T; e_{IS})$ of q-TIP4P/F water, with nuclear quantum effects included (PIMD simulations), obeys the following simple relationship,

$$\mathcal{S}(e_{IS}) = a + b\ e_{IS} \tag{12}$$

where, in equilibrium, $e_{IS} \rightarrow E_{IS}$. a and b are PEL variables that depend on (N, V, T). In this section we validate Eq. 12 for the case of q-TIP4P/F water using PIMD simulations.

To test Eq. 12, we follow the same procedure employed in Ref. [1] to calculate the $\mathcal{S}(e_{IS}, T)$ of the RP-PEL ($N = 512$ and V being constant). Specifically, for a given density ($\rho =$ 0.92, 1.00, 1.16, 1.32 g/cm^3), we first perform classical MD simulations of q-TIP4P/F water at $T' = 200, 210, ..., 400$ K. At each temperature T', we obtain $m = 25$ IS; since we run classical MD simulations at 16 different temperatures, this leads to a total of $n = 400$ IS of the CL-PEL. For each of the $n = 400$ IS, we calculate the corresponding values of e_{IS} and the associated normal mode frequencies $\{\omega_{i,0}\}_{i=1,2,...,9N}$. The so-obtained set of values $(e_{IS}, \{\omega_{i,0}\}_{i=1,2,...,9N})$ quantify the depth and curvature of the IS of the CL-PEL sampled by classical q-TIP4P/F water. Now, it can be shown that an IS of the RP-PEL *with collapsed ring-polymers* is also an IS of the CL-PEL, and vice versa; see Ref. [1]. Moreover, since the ring-polymers are collapsed at the IS of the RP-PEL, both IS (one in the CL-PEL and the other in the RP-PEL) have the same energy e_{IS}. However, the curvature of each IS is different. Specifically, the curvature of the IS at the CL-PEL is given by the normal mode frequencies $\{\omega_{i,0}\}_{i=1,2,...,9N}$, while the curvature of the IS at the RP-PEL is given by the normal mode frequencies $\{\omega_{i,j}\}_{i=1,2,...,9N;j=1,2,...,n_b}$. Both sets of frequencies are related by Eq. 11. Accordingly, we use Eq. 11 and the n sets $(e_{IS}, \{\omega_{i,0}\}_{i=1,2,...,9N})$ associated to the CL-PEL, to extract n sets of IS energy and local curvatures of the RP-PEL, $(e_{IS}, \{\omega_{i,j}\}_{i=1,2,...,9N;j=1,2,...,n_b})$ (*for a given temperature* T of the ring-polymer system since Eq. 11 depends on T). From the so-obtained set of values $(e_{IS}, \{\omega_{i,j}\}_{i=1,2,...,9N;j=1,2,...,n_b})$, one can calculate the shape function $\mathcal{S}(e_{IS}, T)$, for a given density (N and Vconstant) and temperature T.

The blue circles in Fig. S5(a) show the n evaluations of $(e_{IS}, \mathcal{S}(e_{IS}, T))$, by following

the procedure described above, for the case $T = 210$ K and $\rho = 1.00$ g/cm^3 ($N = 512$). The corresponding block-average is indicated by the black circles. The line in the figure is a straight line interpolation of the black circles at approximately $e_{IS} < -56.5$ kJ/mol (these values correspond to $T \leq 280$ K, the temperature range at which the Gaussian approximation holds and E_{IS} decreases upon cooling; see Fig. 1(b) of the main manuscript). It follows that Eq. 12 is in full agreement with our PIMD simulations of q-TIP4P/F water. As shown in Figs. S5(b)-(d), similar results are obtained at $T = 240,\ 270,\ 300$ K and $\rho =$ 1.00 g/cm^3 ($N = 512$). Indeed, we find that Eq. 12 holds at all densities and temperatures studied.

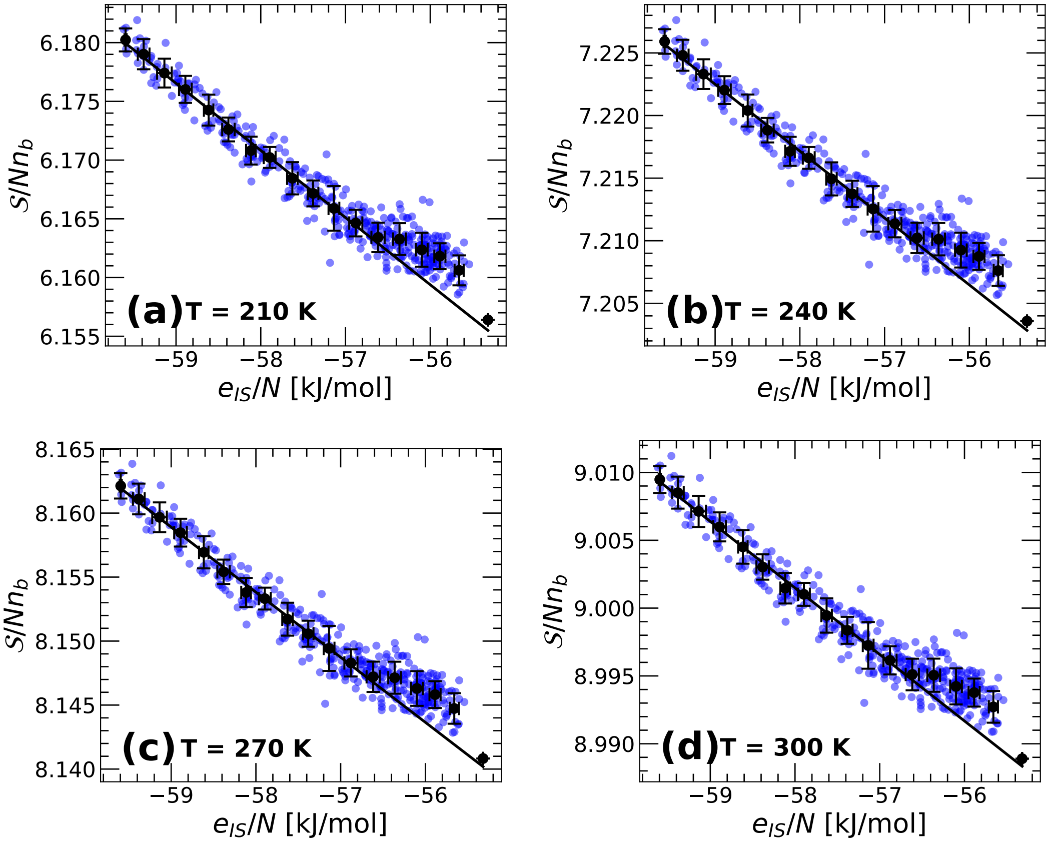

FIG. S5. Shape function of q-TIP4P/F water, including NQE, as a function of the IS energy e_{IS}. The blue circles are the values of $\mathcal{S}(e_{IS}, T)$ obtained by using Eq. 12 (see text) for (a) $T = 210$ K, (b) $T = 240$ K, (c) $T = 270$ K, and (d) $T = 300$ K. The corresponding block-average is indicated by the black circles. Lines are linear fit to the data for $e_{IS} < -56.5$ kJ/mol; these values of e_{IS} correspond to $T \leq 280$ K for which the Gaussian approximation holds for q-TIP4P/F water [see Fig. 1(b) of the main manuscript].

VI. SUPPLEMENTARY NOTE 6

Structural Properties of q-TIP4P/F water at the Inherent Structures

In this section, we focus on the structural properties of q-TIP4P/F water from MD and PIMD simulations at the (i) instantaneous configurations (obtained from MD/PIMD simulations) and (ii) corresponding IS. Target structural properties include radial distribution functions, local order metrics, and the geometrical properties of the hydrogen-bonds between q-TIP4P/F water molecules.

1. Radial distribution functions and local order

Figs. S6(a)-(c) show the OO, OH, and HH radial distribution function (RDF) of q-TIP4P/F water obtained from MD (dashed lines) and PIMD simulations (solid lines) at $T = 240$ K and different densities. At the temperature considered, the RDFs for the classical and quantum q-TIP4P/F water are qualitatively similar but including NQE leads to smoother RDFs, with smaller maxima and shallower minima, relative to the classical RDFs. This means that, consistent with previous studies [15–17], including NQE leads to a slightly less structured q-TIP4P/F water than found in the classical case.

The effects of increasing the density on the RDFs of water are relevant, and qualitatively independent of whether one includes NQE. Both MD and PIMD simulations show that, as the density increases, the OO RDF (i) increases at $r \approx 3.5$ Å, corresponding to the first-interstitial shell of the water molecules, while (ii) it decreases the second maximum located at $r \approx 4.5$ Å. This means that the main effect of increasing the density is to displace the neighboring molecules of a given water molecule from its second hydration shell ($r \approx 4.5$ Å) to its first-interstitial shell ($r \approx 3.5$ Å). In addition, increasing the density reduces slightly the first maximum of the OO RDF located at $r \approx 2.8$ Å, suggesting a distortion of the local tetrahedrality of water with increasing pressure. Overall, the structural changes observed for water at $T = 240$ K with increasing density are consistent with previous computational studies on water [18, 19].

The collapse of the tetrahedral local structure of water with increasing density is also evidenced by the changes in the OH and HH RDFs shown in Figs. S6(c) and S6(d). Upon increasing the density, the first minimum of the OH and HH RDFs increase, as the molecules

get closer together, while the first maximum of the OH and HH RDF decrease slightly. Additional small structural changes are observable in the OH and HH RDFs at $r > 4.0$ Å.

Figs. S6(d)-(f) show the OO, OH, and HH RDFs of q-TIP4P/F water calculated at the IS corresponding to the instantaneous configurations used in Figs. S6(a)-(c). Surprisingly, the RDFs of the quantum (PIMD simulations, solid lines) and classical (MD simulations, dashed lines) practically overlap at all densities considered ($T = 240$ K). This implies that the average structure of q-TIP4P/F water at the IS of the RP-PEL and at the IS of the CL-PEL are practically identical. Since the IS of the RP-PEL of the ring-polymers associated to the O and H atoms of q-TIP4P/F water are collapsed, our results suggest that the IS visited by the quantum version of q-TIP4P/F water (in the RP-PEL), at $T = 240$ K, are the *same* IS visited by the classical q-TIP4P/F water (in the CL-PEL). This is consistent with the very similar values of $E_{IS}(T)$ shown in Fig. 1(b) of the main manuscript at $T = 240$ K for the quantum and classical versions of q-TIP4P/F water (as shown in Fig. S2(a), at this temperature, the quantum and classical versions of q-TIP4P/F water have very similar values of P_{IS} as well). We note, however, that small deviations exist in the values of E_{IS} obtained from classical MD and PIMD simulations, suggesting that at lower temperatures, e.g., $T = 210$ K, the IS visited by the quantum liquid *at a given temperature* are not necessarily the same.

Overall, the structural changes shown in Figs. S6(d)-(f) are consistent with the corresponding changes observed in Figs. S6(a)-(c) and discussed above. As expected, the RDFs of q-TIP4P/F water are more detailed, with sharper maxima and minima, due to the removal of thermal vibrations after minimization of the potential energy of the system. Hence, q-TIP4P/F water is more structured at the IS than at the instantaneous configurations.

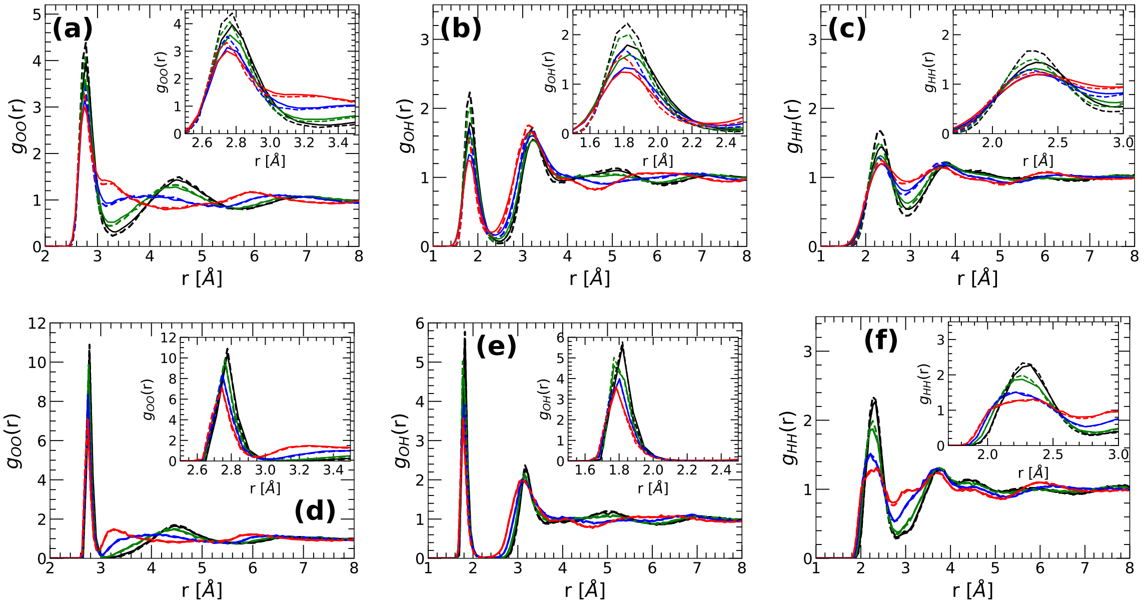

FIG. S6. (a) Oxygen-oxygen, (b) oxygen-hydrogen, and (c) hydrogen-hydrogen radial distribution functions of q-TIP4P/F water obtained from PIMD (solid lines) and classical MD simulations (dashed lines) at $T = 240$ K and selected densities [black, green, blue, and red lines correspond to $\rho = 0.92,\ 1.00,\ 1.16,\ 1.32$ g/cm^3, respectively]. (d)(f)(g) OO, OH, and HH RDF evaluated at the IS corresponding to the instantaneous configurations used in (a)(b)(c). Insets are magnifications of the main panels.

Next, we further characterize the structure of q-TIP4P/F water using a local order metric. Based on the RDFs discussed above, most structural changes in the environment of a given water molecule occur about the molecule's first interstitial shell and second hydration shell. Accordingly, we focus on the the order parameter $\langle d_{fs} \rangle$ defined in [20] which quantifies, approximately, the average separation between the molecules' first and second shells.

In classical MD simulations, the parameter $\langle d_{fs} \rangle$ for a given configuration is defined as follows. For molecule $i = 1, 2, ..., N$, we first define $d_{i,fs}$ as the difference between (i) the distance from the O atom of molecule i to its fifth O neighbor, and (ii) the distance from the O atom of molecule i to its fourth O neighbor. Then, $\langle d_{fs} \rangle$ is defined as the average of $d_{i,fs}$ over all molecules in the system (in equilibrium simulations, one also averages over time). In PIMD simulations, the order parameter $d_{i,fs}$ for molecule i is calculated by averaging over all replicas, i.e.,

$$d_{i,fs} = \frac{1}{n_b} \sum_{k=1}^{n_b} d_{i,fs}^k \tag{13}$$

where $d_{i,fs}^k$ is calculated considering only all O atoms belonging to replica $k = 1, 2, ..., n_b$. $\langle d_{fs} \rangle$ is obtained by averaging of $d_{i,fs}$ over all molecules $i = 1, 2, ..., N$ (and time), as done for the classical case. We note that the minimum value of $\langle d_{fs} \rangle$ for water is 0, corresponding to the full migration of a water molecule from the second hydration shell to the first hydration shell of a given water molecule. Therefore, small values of $\langle d_{fs} \rangle$ imply that water molecules are in a high-density local environment. Large values of $\langle d_{fs} \rangle > 1.0$ Å imply that water molecules are in a low-density arrangement.

Fig. S7 shows the values of $\langle d_{fs} \rangle$ for q-TIP4P/F water obtained from PIMD simulations (solid circles) as a function of temperature and for different densities. Also included are the values of $\langle d_{fs} \rangle$ from MD simulations reported in Ref. [4] (dashed lines). The results from MD and PIMD simulations are qualitatively similar. At a given temperature, $\langle d_{fs} \rangle$ decreases with increasing density indicating that, consistent with the reported changes in the OO RDF, water molecules move from the second shell of a given water molecule, towards its first hydration shell. Interestingly, Fig. S7(c) also shows that $\langle d_{fs} \rangle$ increases monotonically upon cooling. Hence, as the temperature decreases, the second and first shells of the water molecules become increasingly separated. This effect is particular visible at low densities and is consistent with water becoming increasingly tetrahedral upon cooling at approximately $T < 230$ K [15, 21]. We note that the main effect of the inclusion of NQE is to decrease

$\langle d_{fs} \rangle$, for all temperatures and densities. This implies that, again, including NQE leads to a slightly less structure water, relative to the classical case.

The inset of Fig. S7 shows the order parameter $\langle d_{fs} \rangle$ evaluated at the IS of the same configurations used for the main panel of the figure. Not surprisingly, the behavior of $\langle d_{fs} \rangle$ at the IS and instantaneous configurations are very similar to one another. Note, however, that the values of $\langle d_{fs} \rangle$ are slightly larger at the IS since the thermal motion is removed at the IS and the corresponding structure is better resolved than in the instantaneous configurations. Perhaps the most important point from the inset of Fig. S7, is that, at the IS, the values of $\langle d_{fs} \rangle$ obtained from PIMD and classical MD simulations practically overlap at all densities, with small deviations present only at low-density and temperature ($T < 230$ K). Consistent with our previous discussion, this supports the view where the IS visited by the quantum q-TIP4P/F water (in the RP-PEL), at $T > 230$ K, are the same IS visited by the classical q-TIP4P/F water (in the CL-PEL). At low densities, further cooling at $T < 230$ K seems to lead to small differences in the values of $\langle d_{fs} \rangle$ at the IS sampled in the MD and PIMD simulations, suggesting that the corresponding IS are different.

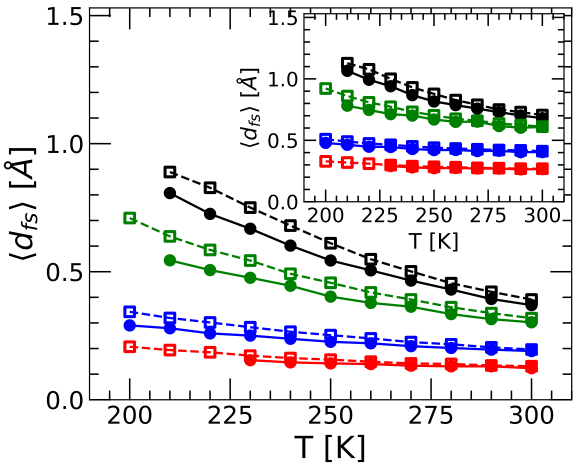

FIG. S7. Average order parameter $\langle d_{fs}(T)\rangle$ for q-TIP4P/F water as a function of temperature and for selected densities [black, green, blue, and red lines correspond to ρ = 0.92, 1.00, 1.16, 1.32 g/cm^3, respectively]. Solid and empty symbols correspond to results obtained from PIMD and classical MD simulations, respectively. Inset: $\langle d_{fs}(T)\rangle$ evaluated at the IS associated to the same configurations used in (a). In all cases, $\langle d_{fs}(T)\rangle$ increases upon isochoric cooling, indicating that the first and second hydration shells of the water molecules become more separated as the temperature decreases. Differences in the values of $\langle d_{fs}(T)\rangle$ from classical MD and PIMD simulations are observable in the instantaneous configurations (main panel); at the IS, values of $\langle d_{fs}(T)\rangle$ are not sensitive to NQE at approximately $T > 230$ K but small differences seem to develop upon cooling at lower densities.

2. Hydrogen-bonds in q-TIP4P/F water

Next, we characterize the hydrogen-bonds (HB) between water molecules at the instantaneous and IS configurations obtained from classical MD and PIMD simulations. We focus on the following properties of the HB, (i) the OO distance between hydrogen-bonded molecules, $d_{OO}^{HB}(T)$, and the (ii) associated HOO angle, $\theta_{HOO}^{HB}(T)$, formed by the O-to-H covalent bond vector (pointing along the O-to-H direction of the donor O atom) and the vector pointing from the donor O atom to the acceptor O atom. In this work, we use the geometrical definition of HB given in Ref. [22] where two water molecules form a HB if the corresponding OO distance is less than 3.5 Å and the HOO angle is less than 30^0.

Fig. S8 shows $\left\langle d_{OO}^{HB}(T)\right\rangle$ and $\left\langle \theta_{HOO}^{HB}(T)\right\rangle$ for q-TIP4P/F water obtained from PIMD simulations (solid circles) as a function of temperature. For comparison, we have also included the results obtained from classical MD simulations (empty squares) reported in Ref. [4]. Results from PIMD and MD simulations are qualitatively similar: $\left\langle d_{OO}^{HB}(T)\right\rangle$ and $\left\langle \theta_{HOO}^{HB}(T)\right\rangle$ decreases with decreasing temperature. In other words, upon cooling, the HB between molecules become slightly shorter and more linear, consistent with liquid water becoming more structured.

An important point from Fig. S8 is the role of NQE on the HB properties. Our MD/PIMD simulations show that at a given density and temperature, introducing NQE leads to slightly longer HB and wider HOO angles. Consistent with the corresponding RDFs and order metric $\langle d_{fs}(T)\rangle$, this implies that the structure of water becomes less pronounced when the atoms delocalizations is included. Overall, our results indicate that the HB network becomes more distorted relative to the classical case.

The insets of Figs. S8(a)(b) show $\left\langle d_{OO}^{HB}(T)\right\rangle$ and $\left\langle \theta_{HOO}^{HB}(T)\right\rangle$ evaluated at the IS sampled by the system in the MD/PIMD simulations. We find that $\left\langle d_{OO}^{HB}(T)\right\rangle$ and $\left\langle \theta_{HOO}^{HB}(T)\right\rangle$ are not sensitive to the inclusion of NQE, except at very low temperatures and for selected densities. At approximately, high-temperatures, Fig. S8 suggests that, again, the classical and quantum system sample the same IS.

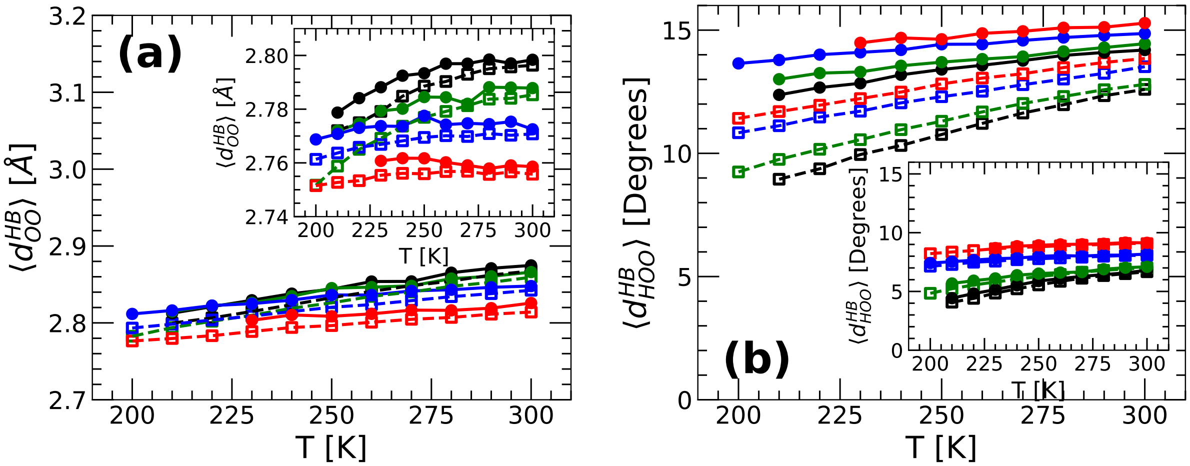

FIG. S8. (a) Temperature-dependence of the average OO distance between q-TIP4P/F water molecules forming a hydrogen-bond, $\langle d_{OO}^{HB}(T) \rangle$. Results are from classical MD (empty squares) and PIMD simulations (solid circles) at densities $\rho = 0.92,\ 1.00,\ 1.16,\ 1.32$ g/cm^3 (black, green, blue, red, respectively). (b) Average HOO angle formed between two hydrogen-bonded water molecules, $\langle \theta_{HOO}^{HB}(T) \rangle$, as a function of temperature. Including NQE (PIMD simulations, solid circles) leads to slightly larger values of both $\langle d_{OO}^{HB}(T) \rangle$ (longer HB length) and $\langle \theta_{HOO}^{HB}(T) \rangle$ (less linear HB), implying that atoms delocalization results in more distorted HBs than found in classical MD simulations. Insets show the $\langle d_{OO}^{HB}(T) \rangle$ and $\langle d_{OO}^{HB}(T) \rangle$ evaluated at the IS.

VII. REFERENCES

[1] Y. Zhou, G. E. Lopez, and N. Giovambattista, J. Chem. Theory Comput. **20**, 1847 (2024).

[2] I. Saika-Voivod, F. Sciortino, and P. H. Poole, Phys. Rev. E **69**, 041503 (2004).

[3] I. Saika-Voivod, P. H. Poole, and F. Sciortino, Nature **412**, 514 (2001).

[4] A. Eltareb, G. E. Lopez, and N. Giovambattista, J. Chem. Phys. **160**, 154510 (2024).

[5] A. Eltareb, G. E. Lopez, and N. Giovambattista, Sci. Rep. **12**, 1 (2022).

[6] P. Gallo, K. Amann-Winkel, C. A. Angell, M. A. Anisimov, F. Caupin, C. Chakravarty, E. Lascaris, T. Loerting, A. Z. Panagiotopoulos, J. Russo, *et al.*, Chem. Rev. **116**, 7463 (2016).

[7] H. B. Callen, *Thermodynamics and an Introduction to Thermostatistics* (John Wiley & Sons, 2006).

[8] A. Eltareb, G. E. Lopez, and N. Giovambattista, J. Chem. Phys. **156**, 204502 (2022).

[9] Y. Liu, G. Sun, A. Eltareb, G. E. Lopez, N. Giovambattista, and L. Xu, Phys. Rev. Res. **2**, 013153 (2020).

[10] B. Nguyen, G. E. Lopez, and N. Giovambattista, Phys. Chem. Chem. Phys. **20**, 8210 (2018).

[11] R. S. Singh, J. W. Biddle, P. G. Debenedetti, and M. A. Anisimov, J. Chem. Phys. **144**, 144504 (2016).

[12] V. Holten and M. Anisimov, Sci. Rep. **2**, 1 (2012).

[13] V. Holten, J. C. Palmer, P. H. Poole, P. G. Debenedetti, and M. A. Anisimov, J. Chem. Phys. **140**, 104502 (2014).

[14] T. E. Gartner, L. Zhang, P. M. Piaggi, R. Car, A. Z. Panagiotopoulos, and P. G. Debenedetti, Proc. Natl. Acad. Sci. **117**, 26040 (2020).

[15] A. Eltareb, G. E. Lopez, and N. Giovambattista, Phys. Chem. Chem. Phys. **23**, 6914 (2021).

[16] S. Habershon, T. E. Markland, and D. E. Manolopoulos, J. Chem. Phys. **131**, 024501 (2009).

[17] J. A. Morrone and R. Car, Phys. Rev. Lett. **101**, 017801 (2008).

[18] A. Eltareb, G. E. Lopez, and N. Giovambattista, Commun. Chem. **7**, 36 (2024).

[19] A. Eltareb, G. E. Lopez, and N. Giovambattista, Phys. Chem. Chem. Phys. **23**, 19402 (2021).

[20] J. Russo and H. Tanaka, Nat. Commun. **5**, 1 (2014).

[21] J. R. Errington and P. G. Debenedetti, Nature (London) **409**, 318 (2001).

[22] A. Luzar and D. Chandler, Phys. Rev. Lett. **76**, 928 (1996).
